# De Novo Green Fluorescent Protein Chromophore-Based Probes for Capturing Latent Fingerprints Using a Portable System

**DOI:** 10.1021/jacs.3c11277

**Published:** 2024-01-08

**Authors:** Nanan Ruan, Qianfang Qiu, Xiaoqin Wei, Jiajia Liu, Luling Wu, Nengqin Jia, Chusen Huang, Tony D. James

**Affiliations:** † The Education Ministry Key Laboratory of Resource Chemistry, Shanghai Key Laboratory of Rare Earth Functional Materials, Shanghai Frontiers Science Research Base of Biomimetic Catalysis, Department of Chemistry, Shanghai Normal University, 100 Guilin Road, Shanghai 200234, China; ‡ Department of Chemistry, University of Bath, Bath BA2 7AY, U.K.; § School of Chemistry and Chemical Engineering, Henan Normal University, Xinxiang 453007, China

## Abstract

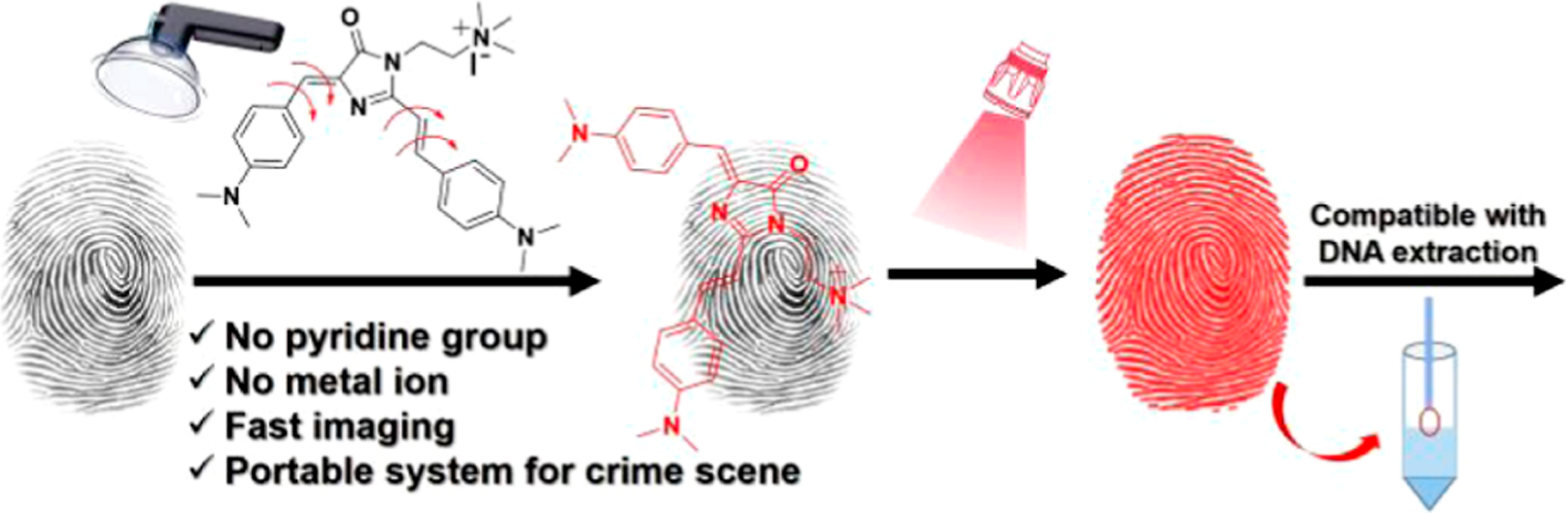

Rapid visualization of latent fingerprints, preferably at their point of origin, is essential for effective crime scene evaluation. Here, we present a new class of green fluorescent protein chromophore-based fluorescent dyes (LFP-Yellow and LFP-Red) that can be used for real-time visualization of LFPs within 10 s. Compared with traditional chemical reagents for LFPs, these fluorescent dyes are completely water-soluble, exhibit low cytotoxicity, and are harmless to users. Level 1–3 details of the LFPs could be clearly revealed through “off–on” fluorescence signal readout. Additionally, the fluorescent dyes were constructed based on an imidazolinone core and so do not contain pyridine groups or metal ions, which ensures that the DNA is not contaminated during extraction and identification after the LFPs are treated with the dyes. Combined with our as-developed portable system for capturing LFPs, LFP-Yellow and LFP-Red enabled the rapid capture of LFPs. Therefore, these green fluorescent protein chromophore-based probes provide an approach for the rapid identification of individuals who were present at a crime scene.

## Introduction

Fingerprints that are formed during an embryo’s development are unique for a particular individual.^1,2^ For example, even identical twins have different fingerprints. Thus, the unique pattern of the fingerprints has been used widely in forensic science for identifying individuals since the late 19th century.^3^ Latent fingerprints (LFPs) are invisible prints formed by sweat or oil that is left on an object after finger contact. The collection of LFPs at crime scenes is an important and widely used operation in forensic science for the identification of individuals.^4^ However, due to their invisible character, the search for LFPs is a significant challenge for forensic investigations. Particularly, given that the rapid capture of LFPs at the crime scene is preferable, the fingerprints will fade over time. To rapidly visualize LFPs, both chemical and physical techniques have been employed, including powder dusting, chemical staining, fuming,^5^ and analysis using electrochemiluminescence imaging,^6^ Raman spectroscopy,^7^ mass spectrometry,^8^ Fourier transform infrared spectroscopy,^9^ and photoacoustic visualization.^10^ However, most of the techniques require expensive and nonportable equipment, which prevents their use at crime scenes for the rapid collection of LFPs. Chemical staining methods have so far prevailed for forensic investigations at crime scenes due to the low cost of the staining reagents and the availability of portable equipment. Thus, various chemical reagent formulations which interact with the components of LFPs have been designed for visualizing LFPs.^11−13^

The primary components of these reagent formulations are chemical dyes, which due to their color and fluorescence properties can facilitate the visualization of LFPs.^14^ Ninhydrin is a typical example since it was first used for developing LFPs in 1954.^15^ Subsequently, many different ninhydrin analogues,^16^ such as 1,8-diazafluoren-9-one,^17^ 1,2-indandione,^18^ and 5-methylthioninhydrin,^19^ have been used for developing LFPs. However, these organic dyes need organic solvents as the cosolvent that can damage biomolecules such as DNA contained in the fingerprints, and the organic solvents are also harmful to the users. Various fluorescent nanomaterials have also been designed for the detection of LFPs.^5,12,20,21^ For instance, carbon dots,^22^ upconversion nanoparticles,^5,23^ and quantum dots^24^ have all been successfully used for developing LFPs due to their water solubility, high photostability, and lower background interference. However, these nanomaterials exhibit intrinsic limitations due to the use of expensive rare earth metals,^23^ toxic heavy metals,^24^ and relatively long visualization time for LFPs (more than 10 min is needed for the development of LFP fluorescent images).^25^ Recently, aggregation-induced emission (AIE) dye-based materials including nanoparticles and powders have been used for developing LFPs.^5^ However, AIE materials also suffer from some limitations. For instance, AIE powders can cause adverse respiratory effects during the dusting process.^26^ In addition, reagent formulations containing AIE materials require harmful organic cosolvents.^12,27,28^ Significantly, some of the AIE dye-based techniques require a washing procedure to remove excess free dye in order to ensure high contrast.^28,29^ Recently, the water-soluble fluorescent probes [TPA-1OH, Zn(tpy-R)] have been prepared for the rapid visualization of LFPs with high contrast.^30,31^ These probes work well on different substrates and can clearly visualize 3-level details of LFPs using a “turn on” mode. However, the probe Zn(tpy-R) needed to be irradiated with UV light (365 nm). In addition, no data are available for the nondestructive collection of DNA from the visualized LFPs after treatment with TPA-1OH or Zn(tpy-R). Since DNA is another unique characteristic of every individual, nondestructive extraction of DNA from LFPs has become a key process following the visualization of LFPs at the crime scene.^20,32^ Significantly, the pyridine group of TPA-1OH and Zn(tpy-R) may interfere with the extraction of DNA from LFPs due to the pyridine-based derivatives exhibiting strong binding affinity toward DNA.^33−35^

**Figure 1:**
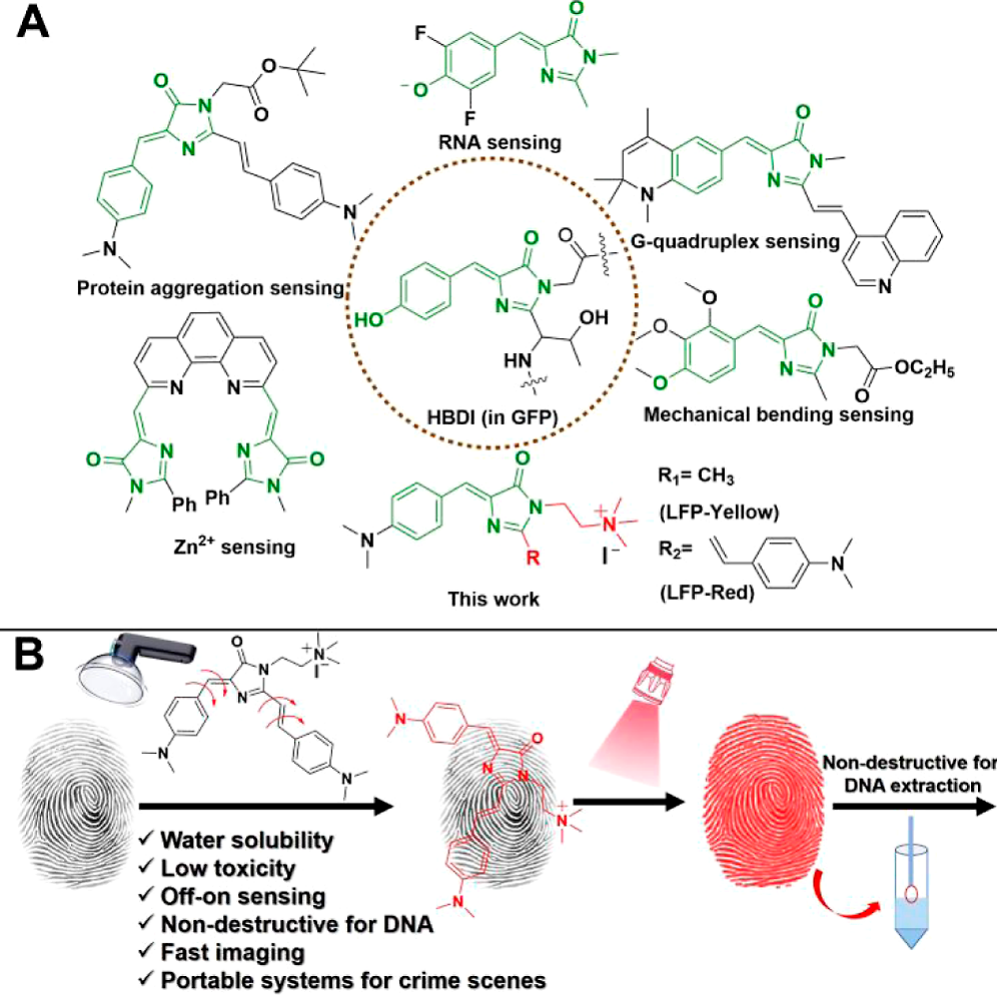
(A) Chemical structures of GFP chromophores developed for sensing. (B) Schematic illustration of the new GFP chromophores designed for visualizing LFPs at crime scenes.

Despite rapid progress, one of the remaining challenges in the field is appropriate portable methodology that can be used for fast visualization of LFPs at the crime scene for forensic investigation.^2^ Additionally, the methodology must be able to produce accurate and high-quality images of the LFPs (preferably producing 3-level details) as well as facilitating the nondestructive collection of DNA samples and they must be nontoxic to the users. Therefore, we now propose imidazolinone-based chemical dyes (LFP-Yellow and LFP-Red, Figure 1) as chemical reagents that can be used in combination with a portable system for the rapid capture of LFPs at crime scenes without DNA destruction. The core structure of LFP-Yellow and LFP-Red is based on imidazolinone which is better known as the green fluorescent protein (GFP) chromophore^36^ formed by amino acids.^37^ GFP chromophore analogues emit bright fluorescence after the torsional motion around the imidazolinone-based part of the molecule becomes restricted, which makes them particularly useful fluorescent dyes since they are easily modified, exhibit high water solubility, and are biocompatibile.^36^ These dyes have found applications in RNA sensing (Figure 1A).^38^ GFP chromophore analogues have also been developed for a large range of applications,^36,39^ including protein aggregation sensing.^40,41^ G-quadruplex sensing,^42^ zinc ion sensing,^43^ and sensing of mechanical bending (Figure 1A).^44^ All of these GFP chromophore analogues exhibit “off–on” fluorescence response due to the restriction of intramolecular motion (RIM)^45^ through the interaction with target molecules. For example, we have successfully extended the use of *p*-hydroxybenzylidene imidazolidinone (HBDI, a typical GFP chromophore) as a fluorescent probe for the “off–on” detection of subcellular viscosity in live cells^46^ due to the RIM of HBDI in high viscosity solutions. Thus, the RIM character of the GFP chromophore analogues led us to hypothesize that the fluorescence of imidazolinone-based dyes could be induced after they were selectively immobilized in certain areas of LFPs (Figure 1B). Significantly, we first disclosed the use of GFP chromophore-based dyes for developing LFPs in 2021^47^ However, details regarding the design strategies, photophysical properties and utility of these dyes have yet to be discussed. As such, with this report, we highlight LFP-Red and LFP-Yellow probes as powerful and practical tools for visualizing LFPs.

## Results and Discussion

### Design Strategy and Synthesis

Chemical techniques for the visualization of LFPs produce images of the ridge pattern of the fingerprint through interaction between the constituents of the LFPs left on the substrates and the chemical reagent formulations. The main chemical constituents of the fingerprints are water, lipids, protein, fatty acids, amino acids, inorganic ions, etc.^48,49^ Herein, we reasoned that the positive charge on the nitrogen of LFP-Yellow and LFP-Red would facilitate binding with the negatively charged fatty acids. Meanwhile, we deduced that any organic molecules with negative charges in the LFPs will also promote anchoring of LFP-Yellow and LFP-Red. To further confirm this hypothesis, we prepared two control probes without positive charges (Scheme S1, Control-Yellow and Control-Red).

**Figure 2:**
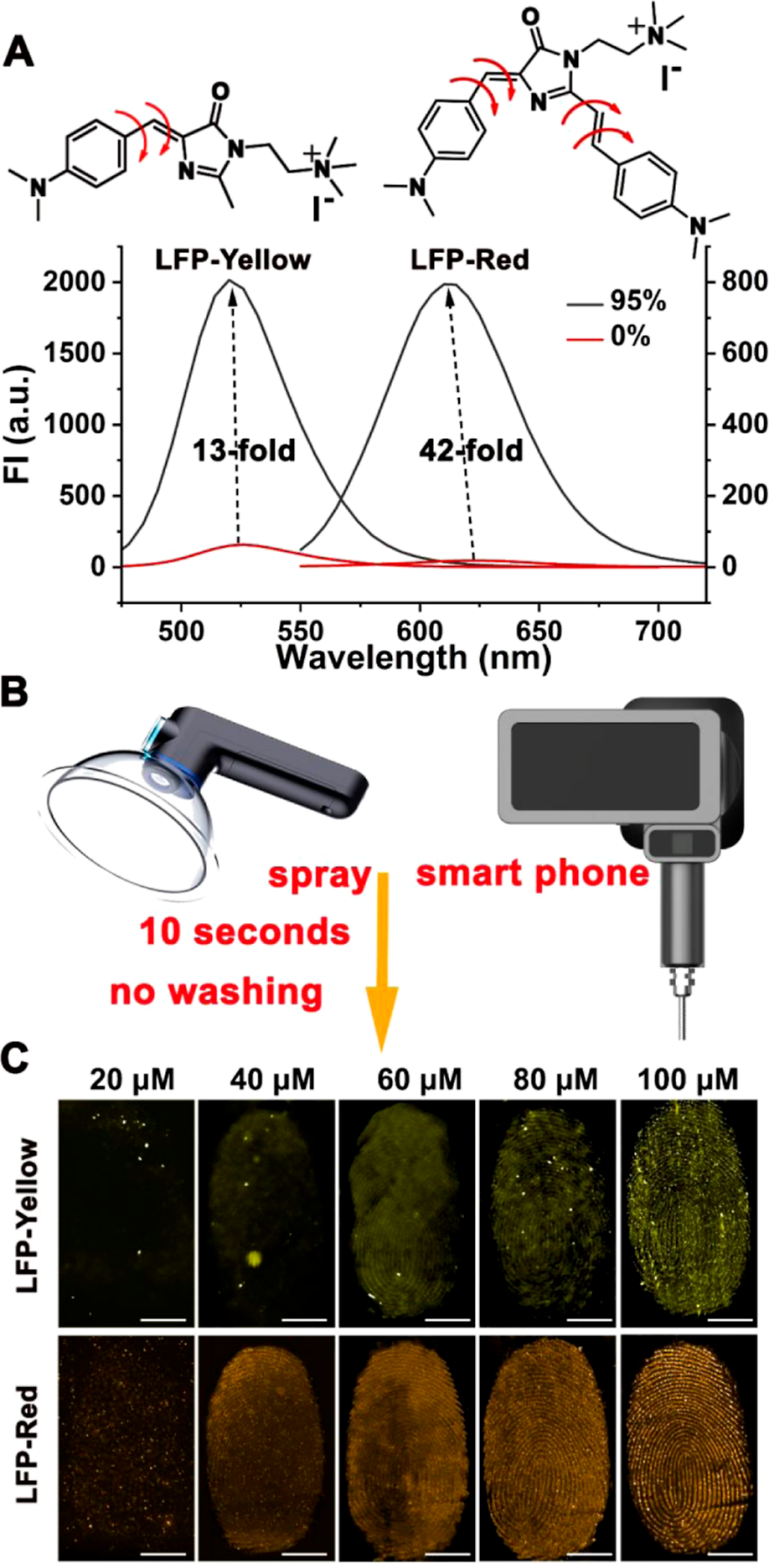
(A) Fluorescence spectra of LFP-Yellow and LFP-Red in pure water (50 μM) with different fractions of glycerol (0% indicated pure water, and 95% indicated the 95 vol % of glycerol in pure water). (B) Design of the portable ultrasonic atomizer for spraying and the portable photographic system for capturing LFPs. (C) RGB color (pseudo color) fluorescent images of LFP on the substrates after spraying with different concentrations of LFP-Yellow (upper for tinfoil) and LFP-Red (bottom for acrylic plate) solution for 10 s, respectively (scale bars: 5 mm, under 445 nm irradiation).

To investigate the water solubility of the target probes (LFP-Red and LFP-Yellow) and control probes (Control-Red and Control-Yellow), the absorption spectra of the probes in pure water with increasing concentrations were explored (Figures S1a, S2a, S3a, and S4a). A good linear relationship between the absorbance and concentration of the probes was observed (Figures S1b, S2b, S3b, and S4b), and this relationship obeys the Beer–Lambert law, suggesting that the probes could be completely dissolved in pure water. Very weak fluorescence for LFP-Red and LFP-Yellow in pure water was observed (Figure 2A, red line). While a remarkable fluorescence enhancement (13-fold for LFP-Yellow and 42-fold for LFP-Red, Figure 2A) was observed when the LFP-Yellow (or LFP-Red) were added to solutions with high viscosity (438.40 cP with 95% glycerol in the pure water). This observation confirmed the RIM characteristics of LFP-Yellow and LFP-Red. The detailed photophysical properties of the two dyes are listed in Tabel S1 (Figures S5–S8). The fluorescence quantum yield changes verified the RIM characteristics of the two dyes. The influence of pH on the fluorescence intensity of LFP-Red and LFP-Yellow were also investigated in solution with low and high viscosity, respectively (Figures S9 and S10). No remarkable fluorescence changes in solutions with low viscosity were observed, and the fluorescence was relatively stable in high-viscosity solutions over a pH range from 6 to 9.

In addition, no fluorescence response of LFP-Yellow or LFP-Red was observed toward common components found in LFPs such as Na^+^, K^+^, Ca^2+^, Cl^–^, and amino acids (Figure S11). We then sought to evaluate the practical use of a LFP-Red (or LFP-Yellow) solution for the visualization of LFPs. When a water solution of LFP-Red was added to tinfoil with LFPs, the soaked LFPs were clearly visualized within 10 s (Movie S1). Clearly, LFP-Red (or LFP-Yellow) were anchored to LFPs inhibiting RIM, resulting in enhanced fluorescence output. Significantly, a very high contrast image was observed, confirming the potential for use at crime scenes. To further facilitate the use of LFP-Red and LFP-Yellow at crime scenes, we developed a portable system including a portable ultrasonic atomizer and photographic system (Figures 2B and S12 and S13). The portable ultrasonic atomizer was equipped with an ultrasonic oscillator. As such, liquid will be converted into microdroplets to form a spray. Using this system solutions of LFP-Red and LFP-Yellow can be nebulized to form a fine mist and cover the LFPs on the target substrate (Movie S2). Compared to a normal portable plastic spray bottle, the ultrasonic atomizer results in a homogeneous and gentle spray that can soak the substrates, preventing the potential destruction of the LFPs from possible splashes when using a normal spray bottle (Figure S14). Despite the fact that ultrasonic nebulizers have already been widely used in commercial applications, the hood of the commercial ultrasonic nebulizers cannot cover the target substrates since most of the commercially available ultrasonic nebulizers were designed for human use and their hoods were prepared just for covering the mouth. As such, we designed and customized a portable ultrasonic nebulizer with a special hood (suitable for covering substrates with a wide range of different shapes), which facilitates an even coverage of the sample by LFP-Red and LFP-Yellow sprayed as a fine mist over the target substrates. The portable photographic system consists of a power supply, irradiation light, and a smart phone for photographing and addressing the images (Figure S13). To verify the system efficiency, the portable system was used for capturing the LFPs on the bottom of a cup (Movie S3). To start with there are no visible fingerprint on the bottom of the cup. After spraying with the LFP-Red solution using the ultrasonic atomizer for 10 s, a very clear fingerprint was captured using the smart phone. These results confirmed that the portable equipment was suitable for rapid visualization of LFPs at crime scenes and suitable for forensic investigation. We next sought to investigate the sensitivity of LFP-Red and LFP-Yellow using our portable system. When LFP-Yellow solution was sprayed as a mist on to LFPs found on tinfoil, the visibility of the LFPs improved with increasing concentration (Figure 2C, upper, grayscale images available from Figure S15). Similarly, a visible and clear fingerprint was observed on an acrylic plate with increasing concentrations of LFP-Red (Figure 2C, below, grayscale images available from Figure S16). We then evaluated the sensitivity, where repeat contacts using the same finger were made with the surface. In this case, each successive fingerprint deposits less residue; as such, the same finger was placed in contact with the surface 5 times, and the LFPs were then evaluated using LFP-Red and LFP-Yellow. The results suggest that LFP-Red and LFP-Yellow could visualize the LFPs even after five repeated contacts, indicating the excellent sensitivity of the two dyes (Figure S17). These results confirmed that LFP-Red (or LFP-Yellow) combined with the portable system produced an effective spray visualization method. Since no significant cytotoxicity of both LFP-Red and LFP-Yellow was observed at concentrations between 70 and 110 μM (Figures S18 and S19), the final concentration of LFP-Red and LFP-Yellow for the spray solution was fixed at 100 μM.

### Visualization of LFPs and Mechanism Exploration

**Figure 3:**
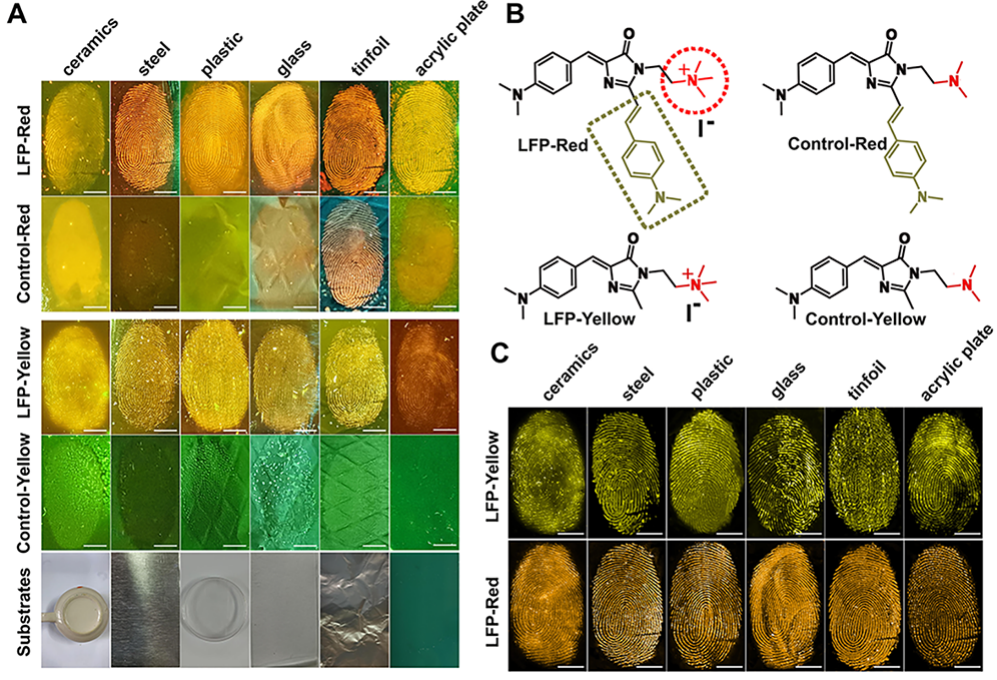
(A) Actual color fluorescent photographs of LFPs on different substrates developed by spraying with an aqueous solution of LFP-Red, Control-Red, LFP-Yellow, and Control-Yellow, respectively. The images were taken by using our photographic system. The concentration of the dyes is 100 μM, and the irradiation wavelength was 445 nm. (B) Chemical structures of the LFP-Red, Control-Red, LFP-Yellow, and Control-Yellow. (C) RGB color fluorescent photographs of LFPs on different substrates developed using ImageJ to remove the color of the substrates (scale bars: 5 mm, under 445 nm irradiation).

Having determined the efficacy of LFP-Red and LFP-Yellow which are a new class of highly efficient fluorescent dyes for visualizing LFPs, we then evaluated the scope of substrates where LFPs can be visualized. Initially we evaluated the visualization of LFPs on typical substrates including ceramics, steel, plastic, glass, tinfoil, and acrylic plate. After the substrates were sprayed with LFP-Red (or LFP-Yellow) solution for 10 s, a clear and high-contrast fingerprint was observed on all the substrates (panel LFP-Red and LFP-Yellow in Figure 3A). Since the substrates have significant effect on the final visualization of the LFPs, different visualization techniques can be used to improve the final quality of the LFPs according to the substrates.^50^ In principle, the portable spraying technique is much more convenient and cost-effective compared with the soaking method. For instance, a significant quantity of solution is needed for visualization of LFPs on a large substrate, such as a car, using the soaking method. However, previously developed spraying methods also have intrinsic limitations compared with the soaking method. Usually, the contrast and resolution of the images of LFPs will be decreased because the solution containing the dyes could not homogeneously interact with the deposited LFPs when using the spraying method. Herein, the utilization of a portable ultrasonic atomizer can address this problem. In addition, from a molecular design point of view, the positive charge on the nitrogen part of LFP-Red and LFP-Yellow improves the interaction between the dyes and deposited LFPs. To probe this hypothesis, control dyes without the positive charge at the nitrogen were prepared (Control-Red and Control-Yellow, Figure 3B), which exhibited similar RIM characteristics as LFP-Red and LFP-Yellow (Figure S20). As anticipated, the control dyes displayed low-resolution imaging of the LFPs on the same substrates with low signal-to-noise (panels Control-Red and Control-Yellow, Figure 3A), which confirmed the importance of the positive charge for the interaction of the LFP-Red and LFP-Yellow with the LFPs. Additionally, to verify that any organic molecules with negative charges such as the fatty acids or DNA in the LFPs can promote anchoring of LFP-Yellow and LFP-Red, we evaluated the efficiency of LFP-Red and LFP-Yellow for LFPs containing negative charged oleic acid and DNA, respectively. The results suggested that LFPs with negative charged oleic acid (or DNA) exhibited stronger fluorescence signals compared to the normal LFPs (Figure S21). Fatty acids are found in the natural secretions from sweat glands under the skin of our fingers, and DNA can be included from the contact of our fingers with the skin or other parts of the body. Therefore, we deduce that fatty acids contribute significantly to the binding between the two dyes and LFPs.^48,49^

To further explore the universality of our method, we compared the visualization of LFPs using 1,2-indanedione (commonly used commercially available reagent for LFPs)^51,52^ and LFP-Red and LFP-Yellow. The results suggest that the 1,2-indanedione cannot visualize LFPs effectively after spraying for 10 s, while LFP-Red (or LFP-Yellow) could visualize the LFPs clearly using just 10 s of spraying (Figure S22). Then, LFP-Red and LFP-Yellow were used for developing LFPs on more difficult surfaces of objects, such as the adhesive side of tapes, decorative surfaces of nonabsorbent objects, rough surfaces of semiadsorbent stones, bricks, and wood (Figure S23). Finally, we extended the practical use of the two dyes by exploring the development of natural LFPs on different substrates from daily life (including a plastic bottle, knife, damaged brick, etc.). As such, LFP-Red and LFP-Yellow solutions were used for visualizing the LFPs generated by the same finger on the surfaces of these substrates (Figure S24). All the results suggest that the LFPs could be effectively visualized.

**Figure 4:**
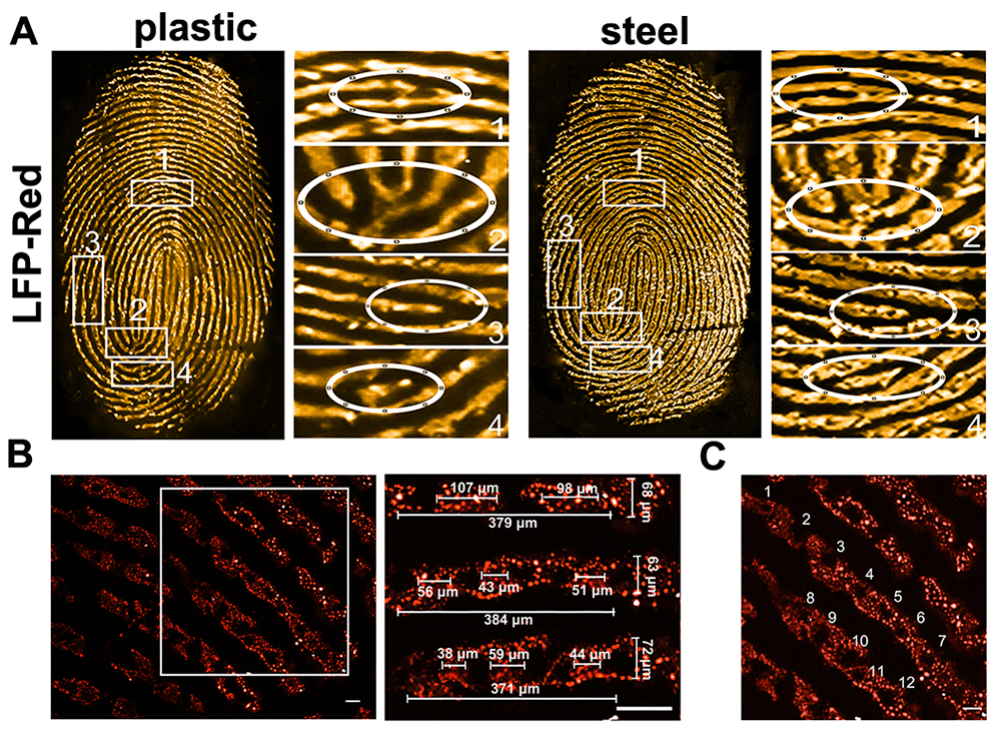
Level 1–3 details of LFP-Red (100 μM)-treated LFPs. (A) Level 1 and Level 2 details of LFPs on plastic and steel were clearly visualized using RGB color (pseudo color) fluorescent images by enlarging the partial regions of the LFPs. Ovals in 1, 2, 3, and 4 insets spotlight the bifurcation, ridge origin, ridge termination, and short ridge, respectively (scale bars: 5 mm, under 445 nm irradiation). (B,C) Level 3 detail was visualized using fluorescent microscopic images for the partial region of LFPs. Scale bar: 100 μm.

Next, we explored the use of LFP-Red and LFP-Yellow for revealing the details of LFPs since the fingerprint features are generally classified into three levels.^14,53^ High contrast and resolution LFPs with clear fluorescent pattern of the morphological information including the overall orientation, pattern type, and focal points of the print whorl on the substrates could be revealed by removing the color of the substrate using ImageJ software (Figures 3C and S25 and S26), indicating clear level 1 detail of the visualized fingerprints. Interestingly, the LFP-Red treated LFPs were of better quality than the LFP-Yellow treated LFPs on all the substrates (Figures S15, S16, S25 and S26). This could be ascribed to a larger π-conjugated system of LFP-Red, which helps decrease the amount of background signal from the substrates. Additionally, the longer chain of LFP-Red makes it much more flexible, which may enable LFP-Red to make multiple contacts with components in the LFPs. Thus, LFP-Red performs better than LFP-Yellow in terms of the resolution and quality of the LFP images. We then selected LFPs on plastic and steel to investigate the ability of LFP-Red and LFP-Yellow to reveal levels 2 and 3 detail. As shown in Figures 4A and S27a, by enlarging the selected regions of the LFPs on the plastic and steel, the bifurcation, short ridge, ridge termination, and ridge origin (level 2 detail) were clearly observed (Figures 4A and S27a, S28, and S29). The level 3 details of fingerprints are microscopic. Using fluorescence microscopy, a partial region of the LFPs was visualized (left panel on Figures 4B and S27b). The specific location, size, and shape of the pores of the LFPs were clearly observed. Specifically, the number, location, and distribution of the pores could be clearly identified using the direct fluorescent pattern under a microscope (Figures 4B,C and S27b,c). The width of different ridges is different, which could be clearly distinguished (right panel on the Figures 4B and S27b). Additionally, the distance between the different sweat pore positions is also different even in the same ridge, which was also clearly observed (right panel of Figures 4B and S27b). These level 1–3 details of the LFPs could provide unique characteristics facilitating the identification of individuals.^54^

### Stability of LFP-Red and LFP-Yellow

**Figure 5:**
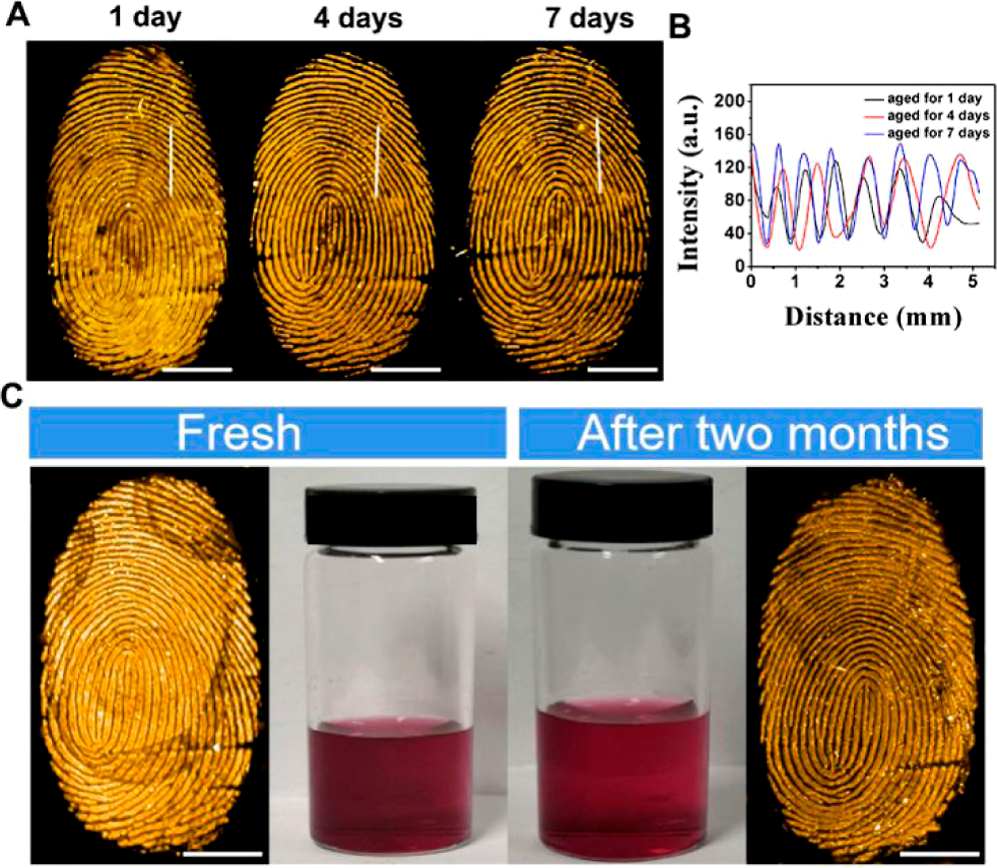
(A) RGB color (pseudo color) fluorescent photographs of LFPs aged for 1, 4, and 7 days on tinfoil are developed by LFP-Red (100 μM) aqueous solution. (B) Variations of the fluorescence intensity between the fingerprint ridge and furrow across the white line. (C) Fresh LFP-Red aqueous solution (100 μM) and after storing for 2 months were used for LFPs development on tinfoil using the spray method (the LFPs are from the same finger) (scale bars: 5 mm, under 445 nm irradiation).

LFPs left on substrates are rarely measured immediately and are often aged for a significant amount of time before being stained, such as in complex crime scenes. Therefore, we evaluated the ability of the reagents for visualizing LFPs after they were aged between 0 and 7 days. The LFPs aged for 1, 4, and 7 days on tinfoil were sprayed with LFP-Red and LFP-Yellow solution, respectively. The obtained fluorescent images displayed no significant difference (Figures 5A and S30a). Additionally, the fluorescence signal of the region of interest is almost identical (Figures 5B and S30b). The results suggest that LFP-Red and LFP-Yellow are suitable for the evaluation of complex crime cases. Also given that the reagents will be used directly at the crime scene, the stability of LFP-Red and LFP-Yellow solutions on storage were determined. After being stored for 2 months, the solutions of the LFP-Red (or LFP-Yellow) remained constant and no significant precipitation was observed (the middle panel in Figures 5c and S30c). In addition, LFPs were still clearly visualized using the 2 month-old solutions compared to the freshly prepared solutions (Figures 5C and S30c). The grayscale images confirmed these results (Figures S31 and S32).

### Evaluation of the Effect of LFP-Red and LFP-Yellow on DNA Identification

**Figure 6:**
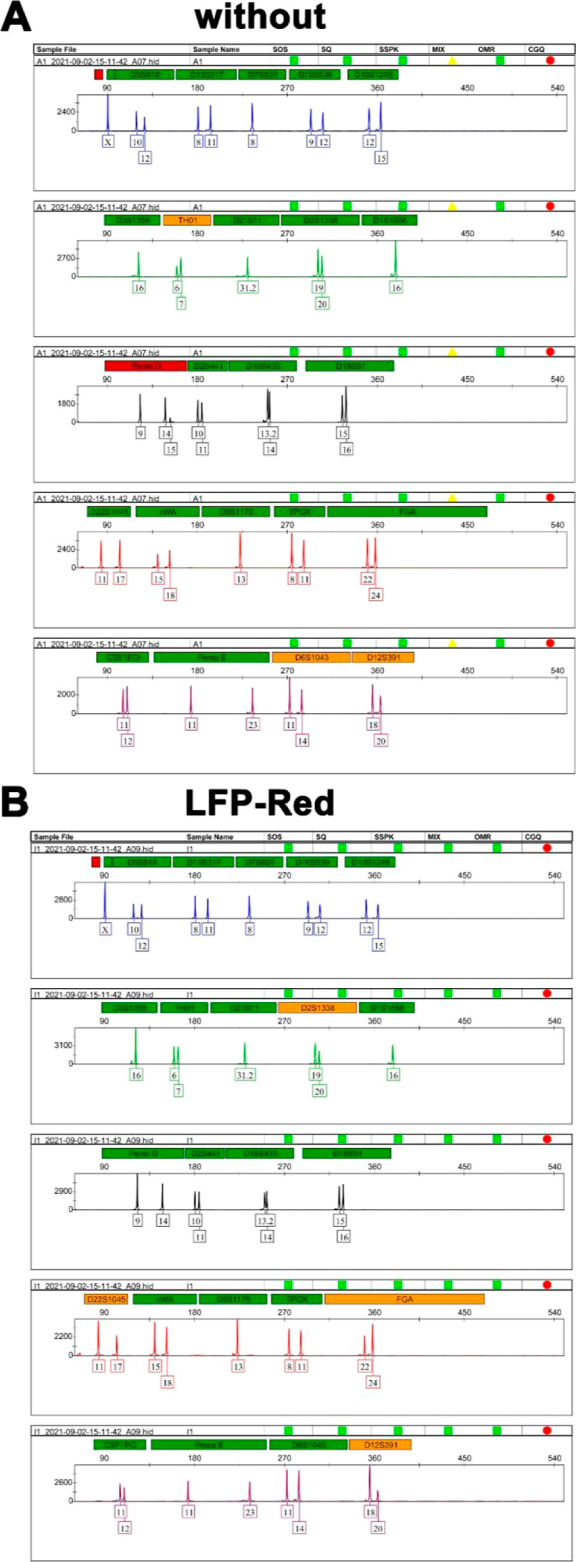
(A) STR analysis of DNA in blood samples. (B) STR analysis of DNA from blood stains after treatment with LFP-Red solution (green: extremely strong genetic locus signal intensity. Orange: strong genetic locus signal intensity. Red: moderate genetic locus signal intensity.).

DNA is another unique characteristic used for the identification of individuals. Therefore, at crime scenes, the nondestructive identification of DNA after revealing the LFPs on substrates is critical for a successful investigation. However, the outstanding challenge for the chemical approach used for LFPs is that the chemical reagents or formulations could generate contamination, making DNA analysis difficult. For instance, chemical reagents or formulations that contain organic solvents, metal ions, and some specific organic compounds including pyridines can potentially interact with DNA, and hence interfere with any subsequent DNA extraction and amplification. Recently developed fluorescent materials have been found to exhibit no toxicity toward cells,^20,21,30,31^ but unfortunately, no investigation on their influence on DNA identification following reading of the LFPs has been reported after using these reagents. Therefore, we used STR (short tandem repeat) analysis to investigate if LFP-Red and LFP-Yellow affect the identification of DNA. Blood stain samples were prepared by mixing LFP-Red and LFP-Yellow solutions with diluted blood, respectively. Then, the double swab technique^55^ was used to collect samples from the blood for DNA analysis. From Figures 6 and S33, there is no difference in the DNA information obtained before and after the blood samples were treated with LFP-Red and LFP-Yellow solutions, respectively. Next, we performed the DNA extraction and detection directly on the surface of LFPs after spraying with a LFP-Red (or LFP-Yellow) solution. The results suggest that no effect was observed on the STR analysis of DNA directly extracted from the LFP-Red (or LFP-Yellow) developed LFPs (Figures S34–S36). Similarly, we conducted the extraction and identification of DNA from the latent blood fingerprints after spraying with LFP-Red and LFP-Yellow, respectively. The results also indicated no effect on the STR analysis (Figures S34–S36). Therefore, these results confirmed the nondestructive extraction of DNA from substrates after LFPs are treated with LFP-Red and LFP-Yellow solutions. Some recently developed chemical reagents have been reported where it was claimed that there was no DNA destruction after being used for LFP visualization; however, no direct data were presented to confirm that conclusion. Herein, we confirmed that LFP-Red and LFP-Yellow exhibited no interference with DNA extraction and identification from the LFPs after they were visualized by LFP-Red and LFP-Yellow solutions, respectively. To the best of our knowledge, this work represents the first example where “off–on” mode fluorescent dyes for LFPs that do not hinder DNA extraction and identification have been reported.

## Conclusions

We developed GFP chromophore-based fluorescent dyes and combined them with a portable detection system for the visualization of LFPs. The results confirmed that LFP-Red and LFP-Yellow could rapidly visualize LFPs (within 10 s) through a fluorescence “off–on” mode. These fluorescent dyes are completely water-soluble and exhibit low cytotoxicity, ensuring that biological samples are not destroyed and that they are not harmful to users. Level 1–3 details of the LFPs could be clearly revealed by these fluorescent dyes through a direct fluorescence signal readout. Additionally, LFP-Red and LFP-Yellow have no pyridinium groups or metal ions that may result in DNA contamination and hinder identification. STR analysis confirmed that no interference was observed when these dyes were used for DNA extraction and identification. Finally, the developed portable apparatus system enabled the rapid capture of LFPs at crime scenes using a spray method. We envision that these probes will be used to effectively develop and collect LFPs at crime scenes. Significantly, the portable system can be further modified by introducing various irradiation light sources with different excitation wavelengths enabling multicolor fluorescence imaging.

## Supporting Information Available

Soaking method for the development of LFPs (MP4)
Portable ultrasonic atomizer for spraying (MP4)
Spraying method with ultrasonic atomizer for the development of LFPs (MP4)
Full experimental materials and procedures for the synthesis of compounds, molecular structures, spectroscopic characterization, and protocols for the development of latent fingerprints (PDF)

## Abbreviations

LFPs: latent fingerprints
GFP: green fluorescent protein
STR: short tandem repeat
