# Supplementary material for: De Novo Green Fluorescent Protein Chromophore-Based Probes for Capturing Latent Fingerprints Using a Portable System
Source: J Am Chem Soc. 2024 Jan 8;146(3):2072–9. doi: 10.1021/jacs.3c11277 (PMC10811623; doi:10.1021/jacs.3c11277)
Supplement: Supplementary file 4 — ja3c11277_si_004.pdf [file ja3c11277_si_004.pdf]

Supporting Information

De Novo Green Fluorescent Protein Chromophore-Based Probes for Capturing Latent Fingerprints Using a Portable System

Nanan Ruan[1‡], Qianfang Qiu[1‡], Xiaoqin Wei[1], Jiajia Liu[1], Luling Wu[1,2]*, Nengqin Jia[1], Chusen Huang[1]*, and Tony D. James[1,2,3]*

1. The Education Ministry Key Laboratory of Resource Chemistry, Shanghai Key Laboratory of Rare Earth Functional Materials, Shanghai Frontiers Science Research Base of Biomimetic Catalysis, Department of Chemistry, Shanghai Normal University, 100 Guilin Road, Shanghai 200234, China. Email: huangcs@shnu.edu.cn.
2. Department of Chemistry, University of Bath, BA2 7AY, UK. E-mail: wllcyl@126.com; T.D.James@bath.ac.uk
3. School of Chemistry and Chemical Engineering, Henan Normal University, Xinxiang 453007, China.
[‡]These authors contribute equally.

Table of Contents

1. Experimental

1.1 Materials and reagents

1.2 Instrumentation

1.3 Synthetic procedures

2. Additional Information

2.1 Probe water solubility

2.2 Photophysical properties of LFP-Red and LFP-Yellow

2.3 Probe selectivity experiments

2.4 Fingerprint imaging device

2.5 Grayscale plot for determining the optimal concentration of the probes and sensitivity of the probes

2.6 Cytotoxicity assay for LFP-Red and LFP-Yellow

2.7 Fluorescence performance of control probes

2.8 Influence of fatty acids and DNA on LFP detection

2.9 Comparison of LFP-Red and LFP-Yellow with 1,2-indanedione

2.10 Scope of surfaces on which LFP can be detected

2.11 RGB and grayscale photographs of LFPs

2.12 Evaluation of the effect of LFP-Red and LFP-Yellow on the identification of DNA

3. NMR Spectra and HRMS

4. Movies for development of LFPs

5. References

1. Experimental

1.1 Materials and reagents

All chemicals and solvents are commercially available and were used without further purification unless otherwise indicated. All the reactions took place in a dry nitrogen atmosphere, and the temperature was measured externally. The synthesized compounds were monitored using thin layer chromatography on silica gel (Hailang, Qingdao, China). Column chromatography was performed on silica gel (200-300 mesh, Qingdao Hailang, China). Minimum Essential Medium was obtained from Hyclone and fetal bovine serum was obtained from Bovogen Biologicals Pty Ltd. The absorption and fluorescence spectra were determined in a quartz test tube (1 cm × 1 cm, volume 3.5 mL). Pure water is ultra-pure water (18 MΩ*cm). The probe solution was prepared by dissolving the probe in ultra-pure water. Probe aqueous solution can be stored at room temperature but should be stored away from light.

1.2 Instrumentation

^{1}H and ^{13}C NMR Spectroscopy. In all experiments, ^{1}H and ^{13}C spectra were recorded using a Bruker AVANCE spectrometer with ^{1}H frequency of 400 MHz, ^{13}C frequency of 101 MHz. ^{1}H shifts were referenced to $CDCl_3$ at 7.26 ppm (or DMSO-d_6 at 2.50 ppm) and ^{13}C shifts were referenced to $CDCl_3$ at 77.16 ppm (or DMSO-d_6 at 39.52 ppm).

High resolution mass spectroscopy. Electrospray ionization (ESI) mass spectra were obtained using an Agilent 6545 Q-TOF.

UV-vis absorption and fluorescence emission spectra. The UV-vis absorption spectra and fluorescence spectra of the compounds were determined using Hitachi U-3900 UV/visible spectrometer and Hitachi F-7000 fluorescence spectrometer, respectively.

Image acquisition equipment for LFPs. The portable apparatus including the ultrasonic atomizer for spraying and photographic system for capturing LFPs were designed by our group. The detailed parameters are semiconductor laser (λ = 445±5 nm, 10 W) for irradiation, Huawei nova 5z smart phone camera (pixel 2340 × 1080) was used for photography, optical filter was optical longpass filter that was used for transmission wavelength more than 500 nm, the cut-off rate is OD5. Detailed design is shown in Figures S12 and S13.

Fingerprint collection. To minimize the impact of LFPs components and provide an environment for better data analysis and comparison, a uniform fingerprint collection process required volunteers to wash their fingers with pure water (ultra-pure water with 18 MΩ*cm), lightly touch their foreheads, and then press their fingers on a selected substrates surface for three seconds. The fingerprint used in this work is the index finger's fingerprint of volunteers. In Figure S21, the index finger was further contacting with DNA (salmon sperm, 1 mg/mL) and Oleic acid (100 μM) aqueous solution, respectively. In Figure S24 where latent fingerprints were formed by contact of same finger directly (the finger was not washed or additional cleaning was performed prior to forming the latent fingermarks) with the surface of substrates including a plastic bottle, knife, magazine, and brick. For the STR analysis, latent blood fingerprints were formed by an index finger that was washed with pure water (ultra-pure water with 18 MΩ*cm), lightly touch their foreheads, and then contacting with a trace blood sample.

.

Human blood. Blood samples from a healthy volunteer were collected by venous puncture without preservatives and conserved with EDTA. The sample was used for DNA analysis and sample collection meets the ethical requirements and is approved by First Affiliated Hospital of Nanjing Medical University.

Substrate selection. Substrates with different surface including tinfoil, steel, ceramics, acrylic plate, glass, plastic, A4 paper, envelope, leather, parcel, magazine, plastic bottle, stone, brick, wood, knife were selected.

Prepared of solution with different concentration of probe. 1 mM stock solution of target probe was prepared in pure water. The different concentration of probe was prepared by diluting the stock solution with pure water.

LFPs was developed with spray method. The aqueous solution with the probe at a concentration was placed into the portable ultrasonic atomizer, then a fine mist was sprayed to cover the target substrates for approximately 10 seconds. The LFPs image was obtained using our portable photographic system (Figure S13).

Real-time fluorescence imaging for LFPs. The portable photographic system was used to real-time record of the LFPs on the ceramics (Movie S3).

LFPs level 3 fingerprint imaging. An inverted fluorescence microscope (OLYMPUS IX71) was used to observe the LFPs formed by LFP-Yellow and LFP-Red aqueous solutions (100 μM) on a cell culture dish (corning petri dish style: 100 mm), and partial images of LFPs were obtained. For LFP-Yellow, excitation filter is 475/35 nm (460-495 nm), and emission filter is 530/50 nm (505-555 nm). For LFP-Red, excitation filter is 530/40 nm (510-550 nm), and emission filter is 605/60 nm (575-635 nm). Using ImageJ, the collected fingerprint images were analyzed at level 3 detail.

Subtraction of background color of the substrates. Since most of the substrates are objects used in daily life, the surfaces of these substrates are not clean, some surfaces are colorful, and even some surfaces are rough. After being developed by LFP-Red or LFP-Yellow, the latent fingerprint images left on these surfaces will exhibit a strong background signal (for instance the background color of the substrates) despite this the visualized latent fingerprints could be clearly observed. To minimize the impact of the surface environment on data analysis for obtaining the optimal test conditions, the background signal was subtracted by ImageJ software. Then the virtual color was used after the subtraction of background of the latent fingerprint images. The detailed process with software ImageJ was as follows: firstly, the actual color fluorescence latent fingerprints images were converted to 8-bit grayscale with the ImageJ software by using the Type command in the Image Menu "Image – Type - 8-bit". Then the background can be subtracted using the "Subtract Background" tool: Process – Subtract background. Then the virtual color was used for the images with the "LUT" tool. The detailed information for process, please see the guideline of ImageJ on the website https://imagej.nih.gov/ij/index.html. In the figure caption, we used the "RGB color fluorescence" to indicate that we used a "virtual color" or "pseudo color" for the images displayed.

1.3 Synthetic procedures

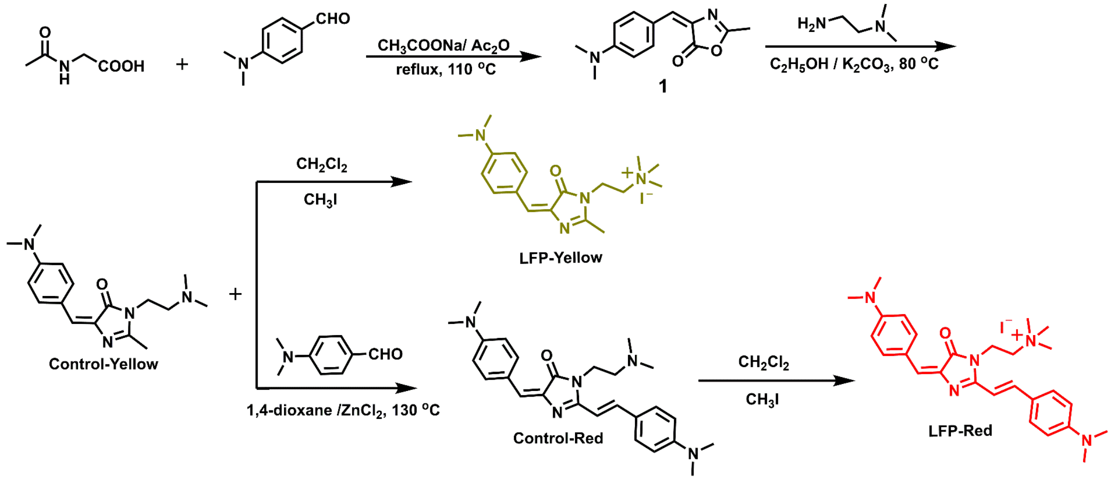

Scheme S1. Synthesis of probes LFP-Yellow and LFP-Red, and control probes Control-Yellow and Control-Red.

Synthesis of compound 1:

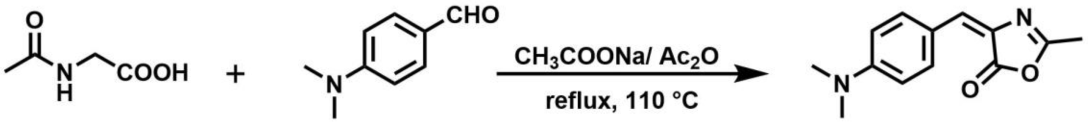

1

Acetylglycine (500 mg, 4.27 mmol), p-dimethylaminobenzaldehyde (530 mg, 3.56 mmol), sodium acetate (437 mg, 5.33 mmol) and acetic anhydride (6 mL) were added to a 100 mL round bottom flask. The mixture was stirred at 110 °C under a nitrogen atmosphere for 3-4 hours. After cooling to room temperature (about 25 °C), precipitates appeared, and 15 mL n-hexane was added. The precipitate was then filtered, and the obtained filtrate cake was rinsed with cold ethanol. Then the product was dried overnight in a vacuum drying oven at 60 °C to provide the compound **1** as dark red solid (533 mg, 65%). The product was pure enough for the next synthesis. ^{1}H NMR (400 MHz, $CDCl_3$) δ 8.00 (d, J = 8.9 Hz, 2H), 7.09 (s, 1H), 6.70 (d, J = 8.9 Hz, 2H), 3.08 (s, 6H), 2.37 (s, 3H). ^{13}C NMR (101 MHz, $CDCl_3$) δ 168.91, 163.00, 152.27, 134.56, 133.01, 127.71, 121.40, 111.81, 40.15, 15.69.

Synthesis of compound Control-Yellow:

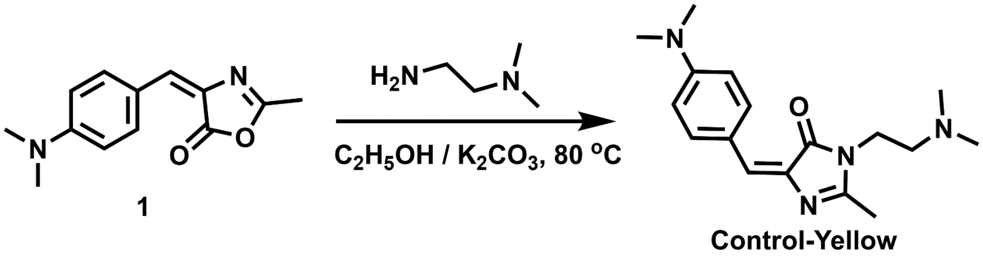

To a solution of compound **1** (500 mg, 2.17 mmol) in absolute ethanol (15 mL), *N*, *N*-dimethylethylenediamine (474 μL, 4.34 mmol), and potassium carbonate (495 mg, 3.58 mmol) were added. The mixture was stirred at 80 °C under a nitrogen atmosphere for 10 hours. After removal of the solvent, the residue was purified by flash chromatography on silica gel using CH_2Cl_2/CH_3OH (v/v, 20/1) as eluent to afford Control-Yellow as a yellow solid (292 mg, 45%). ^1H NMR (400 MHz, DMSO-d_6) δ 8.05 (d, J = 8.6 Hz, 2H), 6.85 (s, 1H), 6.73 (d, J =9.0 Hz, 2H), 3.63 (t, J = 6.4 Hz, 2H), 2.99 (s, 6H), 2.40(t, J = 6.5 Hz, 2H), 2.35 (s, 3H), 2.18 (s, 6H). ^{13}C NMR (101 MHz, DMSO-d_6) δ 169.63, 159.99, 151.18, 134.50, 133.79, 126.50, 121.65, 111.56, 57.55, 54.87, 45.19, 37.90, 15.28.

Synthesis of compound Control-Red:

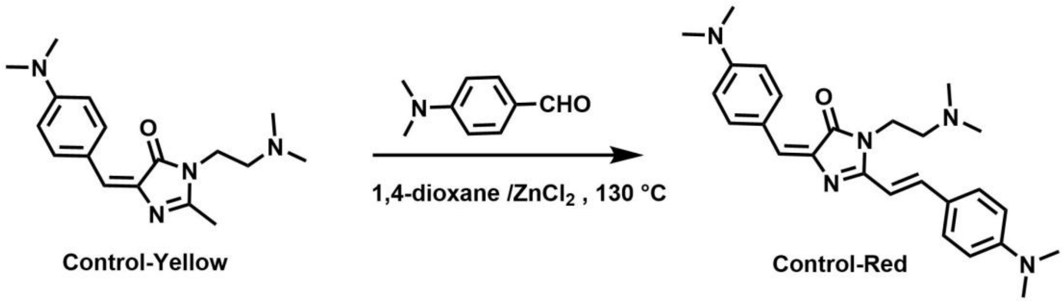

A solution of Control-Yellow (500 mg, 1.6 mmol), p-dimethylaminobenzaldehyde (358 mg, 2.4 mmol), and zinc chloride (2.2 g, 16 mmol) in dioxane (20 mL) was stirred at 130 °C under a nitrogen atmosphere for 2-4 hours. After removal of the solvent, the residue was purified by flash chromatography on silica gel using methylene chloride/methanol (CH_2Cl_2/CH_3OH, v/v, 25/1) as eluent to afford Control-Red as a red solid (414 mg, 60%). ^1H NMR (400 MHz, DMSO-d_6) δ 8.14 (d, J = 8.4 Hz, 2H), 7.82 (d, J = 15.5 Hz, 1H), 7.63 (d, J = 8.5 Hz, 2H), 6.85 (d, J = 15.5 Hz, 1H), 6.81 (s, 1H), 6.79-6.74 (m, 4H), 3.83 (t, J = 6.3 Hz, 2H), 3.02 (s, 6H), 2.99 (s, 6H), 2.40 (t, J = 6.4 Hz, 2H), 2.17 (s, 6H). ^{13}C NMR (101 MHz, DMSO-d_6) δ 169.91, 157.55, 151.44, 151.09, 139.24, 135.66, 133.88, 133.02, 129.77, 124.89, 123.01, 122.49, 112.00, 111.86, 110.81, 108.14, 58.20, 45.44, 45.35, 39.82, 39.73, 37.44. (Note some signals for the carbons are hidden by the solvent).

Synthesis of probe LFP-Yellow

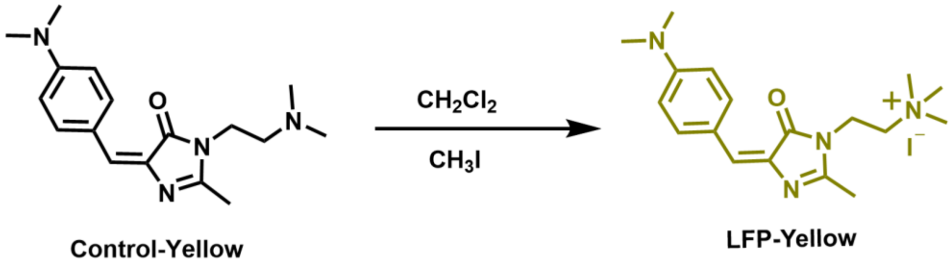

Control-Yellow (300 mg, 1 mmol) was dissolved in 150 mL methylene chloride (CH_2Cl_2), and the reaction mixture was heated to dissolve the solid completely. Then, methyl iodide (1.7 mL, 27 mmol) was added to the solution which was stirred at 25 ºC for 48 hours. The crude product was obtained by centrifugation. Then the crude product was resuspended in CH_2Cl_2 and centrifugated again 2 times to provide the final pure product which is dark yellow solid powder (245mg, 55%). ^{1}H NMR (400 MHz, DMSO-d_6) δ 8.07 (d, J = 8.7 Hz, 2H), 6.93 (s, 1H), 6.74 (d, J = 9 Hz, 2H), 4.05 (t, J = 7.3 Hz, 2H), 3.60 (t, J = 7.3 Hz, 2H), 3.19 (s, 9H), 3.00 (s, 6H), 2.43 (s, 3H). ^{13}C NMR (101 MHz, DMSO-d_6) δ169.40, 158.63, 151.55, 134.12, 133.49, 127.85, 121.20, 111.68, 62.07, 52.53, 39.64, 33.80, 15.42. HRMS (ES+, m/z): calced for $C_{18}H_{27}N_4O^+$: 315.2179, found: 315.2186.

Synthesis of probe LFP-Red

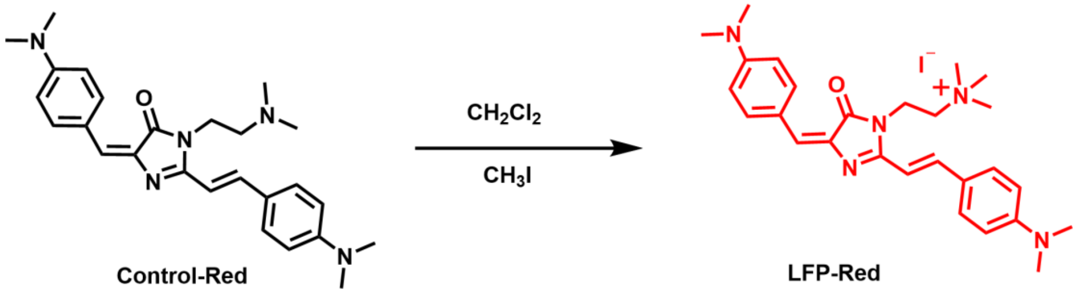

Control-Red (100 mg, 0.23 mmol) was dissolved in 80 mL CH_2Cl_2 and heated until it was completely dissolved. Methyl iodide (0.57 mL, 6.26 mmol) was added to the solution which was stirred at 25°C for 48 hours. The crude product was obtained by centrifugation. Then the crude product was resuspended in CH_2Cl_2 and centrifugated again 2 times to provide the final pure product which is black solid powder (82 mg, 62%). ^{1}H NMR (400 MHz, DMSO-d_6) δ 8.16 (d, J = 8.5 Hz, 2H), 7.88 (d, J = 15.2 Hz, 1H), 7.73 (d, J = 9.3 Hz, 2H), 6.94 (d, J =14.7 Hz, 1H), 6.88 (s, 1H), 6.79 – 6.73 (m, 4H), 4.26 (t, J = 7.4 Hz, 2H), 3.64 (t, J = 7.3 Hz, 2H), 3.23 (s, 9H), 3.03 (s, 6H), 3.00 (s, 6H). ^{13}C NMR (101MHz, DMSO-d_6) δ 169.67, 156.16, 151.53, 151.51, 151.31, 151.30, 140.16, 134.78, 134.14, 130.27, 125.91, 122.91, 122.18, 111.86, 111.82, 107.25, 62.77, 52.53, 33.56. (Note some signals for the carbons are hidden by the solvent). HRMS (ES+, m/z): calced for $C_{27}H_{36}N_5O^+$: 446.2914, found: 446.2921.

2. Additional Information

2.1 Probe water solubility

To evaluate the aqueous solubility of the probes, we measured the UV-Vis absorption spectra of the probes LFP-Red, LFP-Yellow and their control probes Control-Red, Control-Yellow at different concentrations (10 μM, 20 μM, 30 μM, 40 μM, 50 μM, 60 μM, 70 μM, 80 μM, 90 μM, 100 μM) and linearly fitted the probe concentrations with the corresponding UV-Vis absorptions. From Figure S1-Figure S4, it can be seen that the absorption of the probes LFP-Red, LFP-Yellow, Control-Red, and Control-Yellow increased with increasing concentrations, and the values of the absorption and concentration values were linearly related, and the coefficients of determination R^2 of their linear curves were 0.9994, 0.9998, 0.9974, and 0.9964, indicating that the probes LFP-Red and LFP-Yellow and their control probes Control-Red and Control-Yellow exhibit good water solubility over this concentration range.

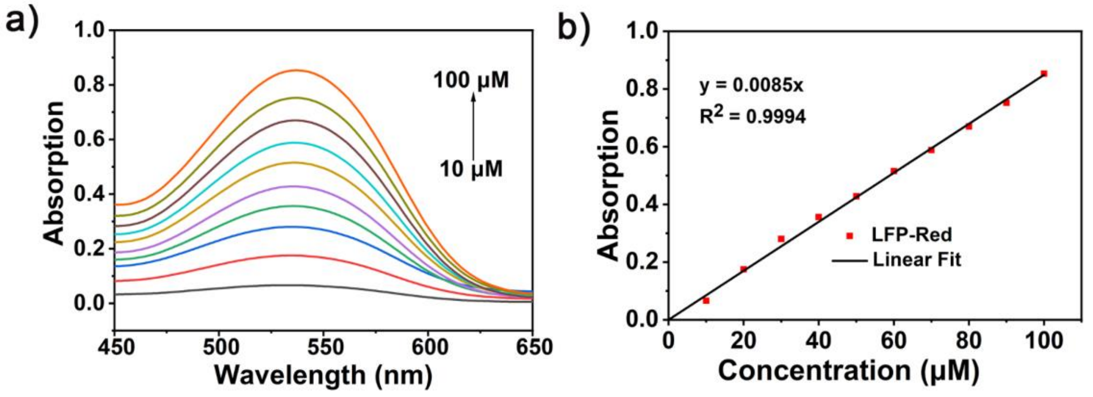

Figure S1. (a) The absorption spectra of LFP-Red in water with gradually increasing concentrations from 10 μM to 100 μM. (b) Relationship between absorbance at 538 nm in (a) with the concentration of LFP-Red in water.

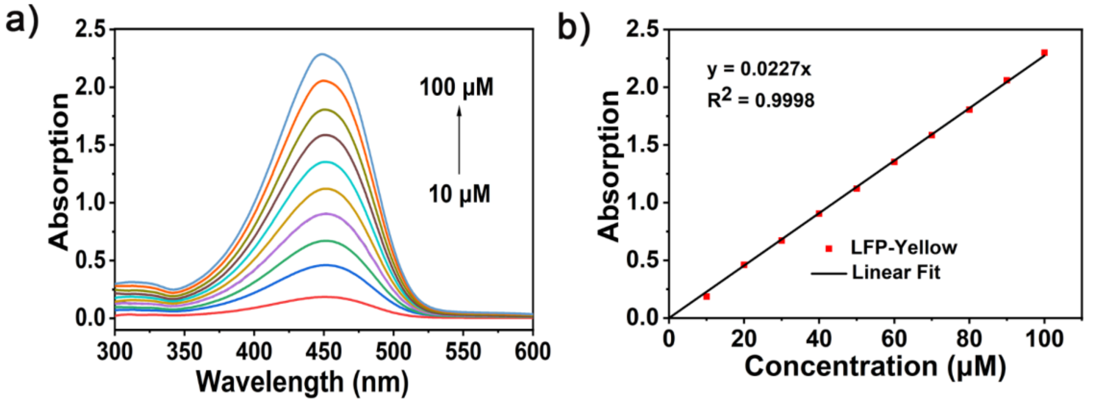

Figure S2. (a) The absorption spectra of LFP-Yellow in water with gradually increasing concentrations from 10 μM to 100 μM. (b) Relationship between absorbance at 453 nm in (a) with the concentration of LFP-Yellow in water.

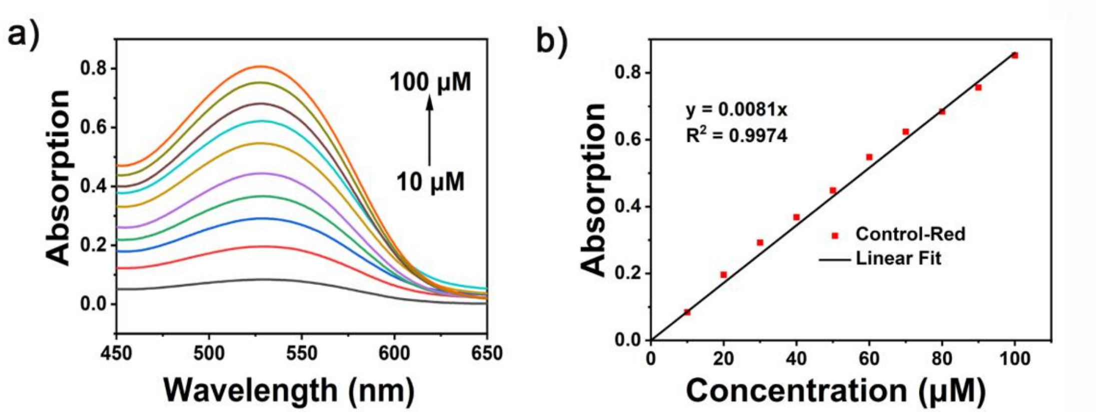

Figure S3. (a) The absorption spectra of control probe Control-Red in water with gradually increasing concentrations from 10 μM to 100 μM. (b) Relationship between absorbance at 538 nm in (a) with the concentration of Control-Red in water.

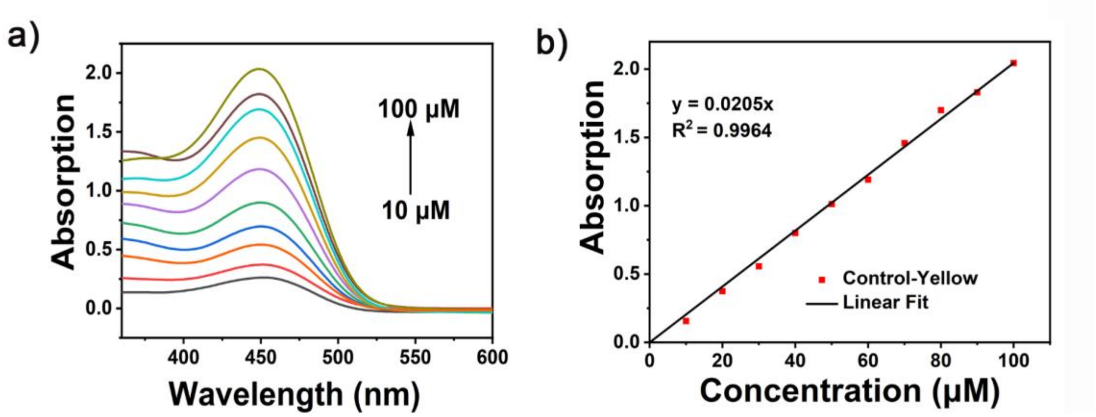

Figure S4. (a) The absorption spectra of control probe Control-Yellow in water with gradually increasing concentrations from 10 μM to 100 μM. (b) Relationship between absorbance at 453 nm in (a) with the concentration of Control-Yellow in water.

2.2 Photophysical properties of LFP-Red and LFP-Yellow

Table S1. Photophysical properties of LFP-Red and LFP-Yellow.

	Viscosity (cP)	$\lambda_{max\ abs}$ (nm)	ε ($M^{-1}cm^{-1}$)	$\lambda_{max\ em}$ (nm)	Φ^a
LFP-Red	0.89	538	8 500	623	0.002
	438.40		10 300		0.120
LFP-Yellow	0.89	453	22 700	526	0.007
	438.40		23 100		0.118

[a]**MIT-V** in high viscosity (438.4 cP, with 95% glycerol in the pure water) at 25°C as standard (Φ = 0.13)[1] for LFP-Yellow. **Cy5** in pure water at 25°C as standard (Φ = 0.28)[2] for LFP-Red.

The relative fluorescence quantum yields (Φ) were measured on optically dilute samples (absorbance < 0.05) which were degassed by bubbling with oxygen-free nitrogen.

$$\Phi_{sample} = \Phi_{standard}\left(\frac{Grad_{sample}}{Grad_{standard}}\right)\left(\frac{\eta_{sample}^2}{\eta_{standard}^2}\right)$$

Where Φ (sample) is the relative fluorescence quantum yield of LFP-Red and LFP-Yellow in a solution with low and high viscosity, respectively. And two fluorescent dyes were taken as the standard. They are **MIT-V** and **Cy5**. **MIT-V** was previously reported by our group[1], and it was used as the standard for testing the fluorescence quantum yield of LFP-Yellow. The fluorescence quantum yield of **MIT-V** in high viscosity (438.4 cP, with 95% glycerol in the pure water) at 25°C is 0.13[1]. The Cy5 was used as the standard for testing the fluorescence quantum yield of LFP-Red. And the fluorescence quantum yield of **Cy5** in pure water is 0.28[2]. Φ(standard) is the fluorescence quantum yield of **MIT-V** in high viscosity (438.4 cP, with 95% glycerol in the pure water) and **Cy5** in pure water, respectively. Grad (standard) and Grad (sample) are the gradients from the plot of integrated fluorescence intensity *vs* absorbance (absorbance < 0.05). Grad (standard) of **Cy5** was calculated to be 16122, Grad (standard) of **MIT-V** was calculated to be 9180, Grad (sample) of LFP-Red were calculated to be 139 and 6918 and Grad (sample) of LFP- Yellow were calculated to be 482 and 7171 for solutions with low viscosity (0.89 cP) and high viscosity (438.4 cP), respectively. The $\eta_{standard}$ and η_{sample} are the refractive indexes of the solvents. Where, $\eta_{standard}$ is 1.333 (for water) and 1.466 (for water/ glycerol solution with viscosity of 438.4 cP). The η_{sample} is 1.333 (for water) and 1.466 (for water/ glycerol solution with viscosity of 438.4 cP). The refractive index of the solvents was determined using an Abbe refractometer (WYA-2S, Shanghai INESA) at 25 ºC.

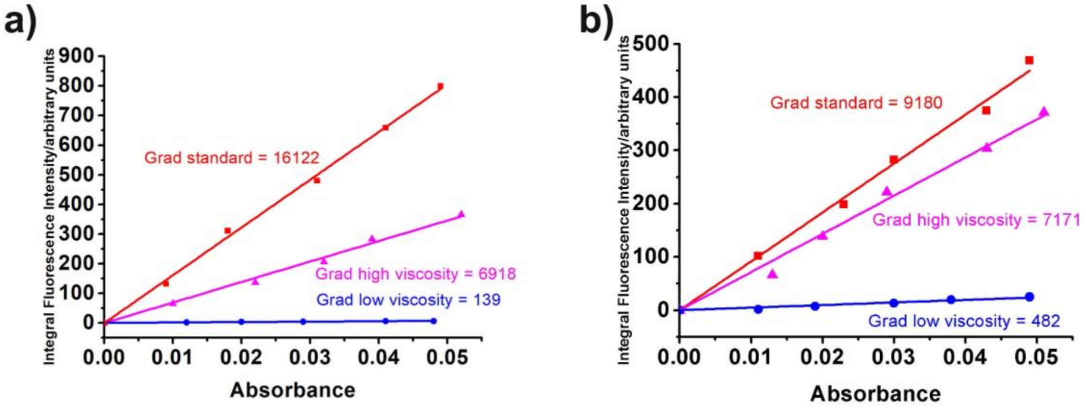

Figure S5. Plots to help determine the fluorescence quantum yields for LFP-Red (a) and LFP-Yellow (b) in buffer with low and high viscosity, respectively.

The molar absorption coefficients (ε) of LFP-Red and LFP-Yellow were obtained using the Beer–Lambert law. Thus, the formula for Beer–Lambert law was A = εlc. Where, A was the maximum absorption of LFP-Red or LFP-Yellow, ε was the molar absorption coefficient, l was the path length of the cuvette (1 cm), and c was the concentration of LFP-Red or LFP-Yellow. Therefore, by measuring the intensity of absorption and varying the concentration of LFP-Red and LFP-Yellow in solution, we obtained concentration versus absorbance plots (Figure S1, Figure S2, Figure S6, Figure S7). In low viscosity, the probe LFP-Red has a slope of 0.0085 and the probe LFP-Yellow has a slope of 0.0227. In high viscosity, the probe LFP-Red has a slope of 0.0103 and the probe LFP-Yellow has a slope of 0.0231. In low viscosity, the molar absorption coefficient (ε) of LFP-

Red was calculated to be 8500 $M^{-1}cm^{-1}$, while in high viscosity, it was 10300 $M^{-1}cm^{-1}$. Similarly, the molar absorption coefficient (ε) of LFP-Yellow was determined to be 22700 $M^{-1}cm^{-1}$ in low viscosity and 23100 $M^{-1}cm^{-1}$ in high viscosity.

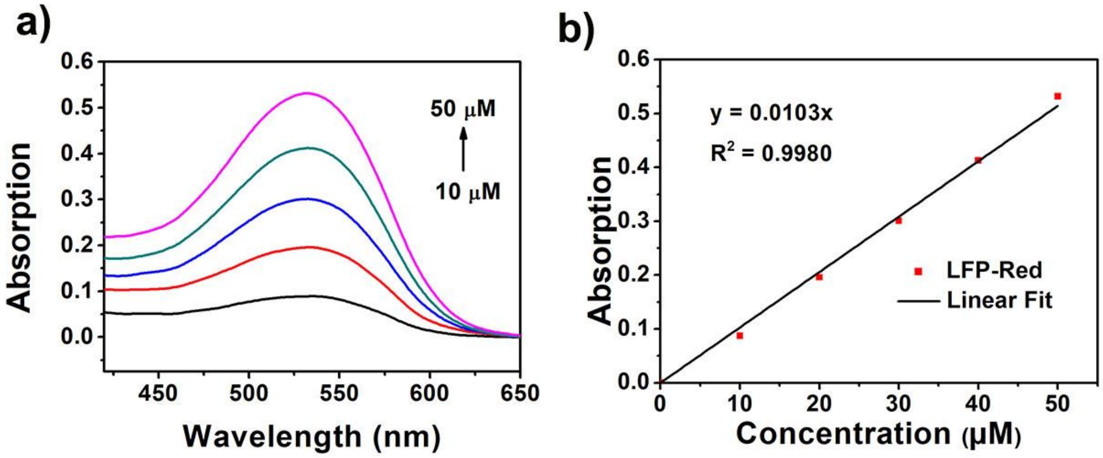

Figure S6. (a) The absorption spectra of LFP-Red in high viscosity (438.4 cP, with 95% glycerol in the pure water) with gradually increasing concentrations from 10 μM to 50 μM. (b) Relationship between absorbance at 538 nm in (a) with the concentration of LFP-Red in high viscosity.

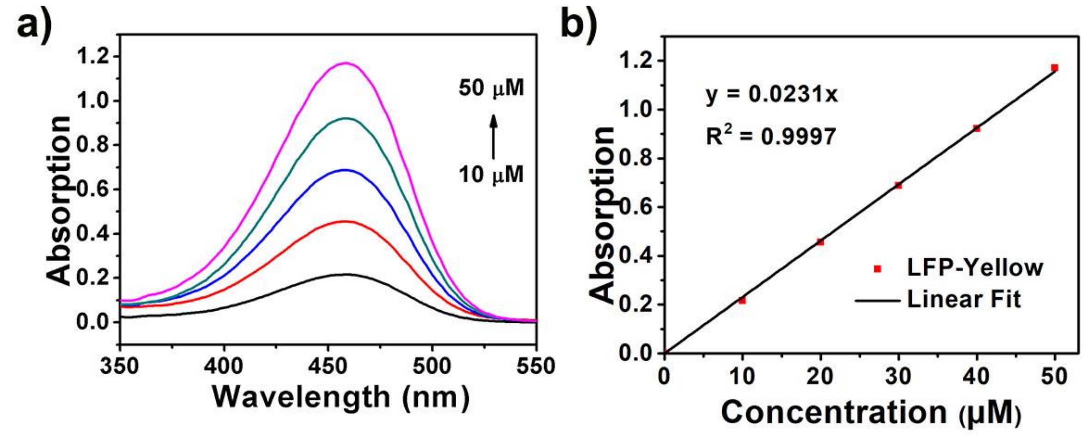

Figure S7. (a) The absorption spectra of LFP-Yellow in high viscosity (438.4 cP, with 95% glycerol in the pure water) with gradually increasing concentrations from 10 μM to 50 μM. (b) Relationship between absorbance at 453 nm in (a) with the concentration of LFP-Yellow in high viscosity.

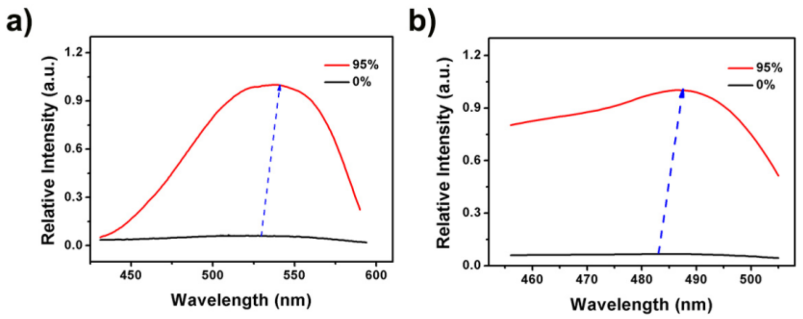

Figure S8. (a) Excitation spectra of LFP-Red in pure water (50 μM) with different fractions of glycerol (0% indicated pure water, and 95% indicated the 95 percent of glycerol in pure water) and fixed emission wavelength of λ_{em} = 623 nm. (Slit width ex = 10 nm and em = 10 nm; temperature 25°C). (b) Excitation spectra of LFP-Yellow in pure water (50 μM) with different fractions of glycerol (0% indicated pure water, and 95% indicated the 95 percent of glycerol in pure water) and fixed emission wavelength of λ_{em} = 526 nm. (Slit width ex = 10 nm and em = 10 nm; temperature 25°C).

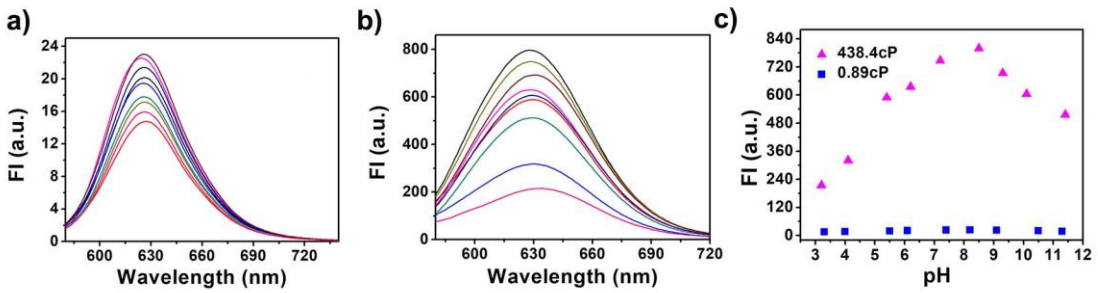

Figure S9. pH dependent fluorescence changes of LFP-Red (50 μM) in pure water (a) and 95% glycerol in the pure water (b) with the pH changes from 3 to 11. (c) Effect of pH on the maximum fluorescence intensity of LFP-Red at low (0.89 cP, in pure water) and high (438.4 cP, 95% glycerol in pure water) viscosity. (λ_{ex} = 538 nm. Slit width ex = 10 nm and em = 10 nm; temperature 25°C).

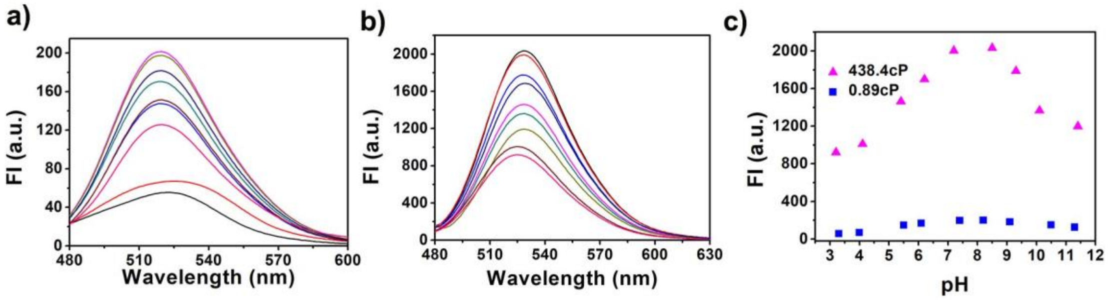

Figure S10. pH dependent fluorescence changes of LFP-Yellow (50 μM) in pure water (a) and 95% glycerol in pure water (b) with pH changes from 3 to 11. (c) Effect of pH on the maximum fluorescence intensity of LFP-Yellow at low (0.89 cP, in pure water) and high (438.4 cP, 95% glycerol in the pure water) viscosity. (λ_{ex} = 453 nm. Slit width ex = 10 nm and em = 10 nm; temperature 25°C).

2.3 Probe selectivity experiments

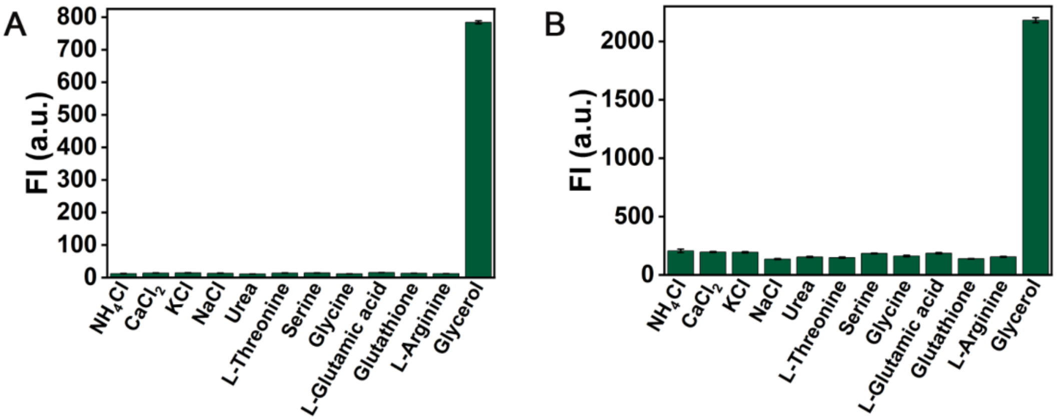

Figure S11. (A) The maximum fluorescence intensity (623 nm) of LFP-Red (50 μM) in different amino acids (20 mM), salt solutions (20 mM) and glycerol (438.4 cp). (B) The maximum fluorescence intensity (526 nm) of LFP-Yellow (50 μM) in different amino acids, salt solutions (20 mM) and glycerol (438.4 cp). (λ_{ex} = 538 nm, λ_{em} = 623 nm for LFP-Red, λ_{ex} = 453 nm, λ_{em} = 526 nm for LFP-Yellow. Slit width ex = 10 nm and em = 10 nm; temperature 25°C).

2.4 Fingerprint imaging device

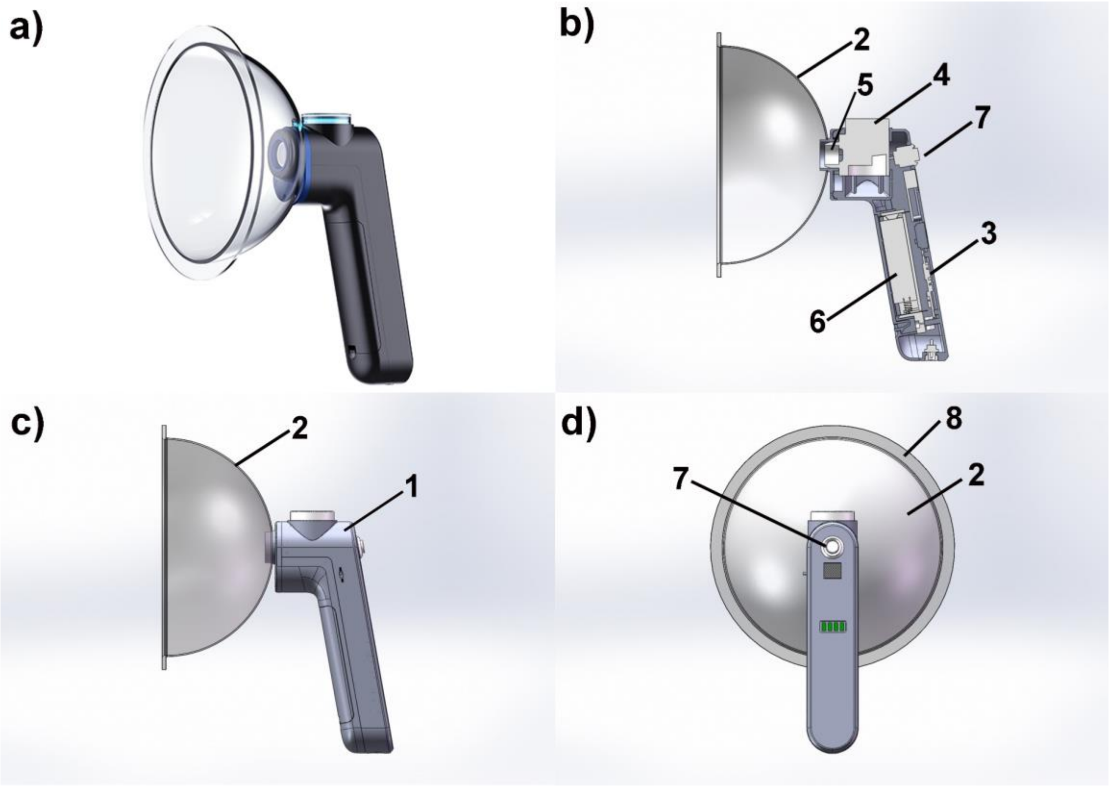

Figure S12. Schematic diagram of the portable ultrasonic atomizer for spraying. a) Actual portable ultrasonic atomizer. b) The internal structural diagram of the portable ultrasonic atomizer. c) Side view of portable ultrasonic atomizer. d) Back view of portable ultrasonic atomizer. 1: main body of the ultrasonic atomizer. 2: spray hood. 3: motherboard. 4: small liquid storage tank. 5. ultrasonic oscillator. 6: lithium battery. 7: control button. 8: outer edge of spray hood.

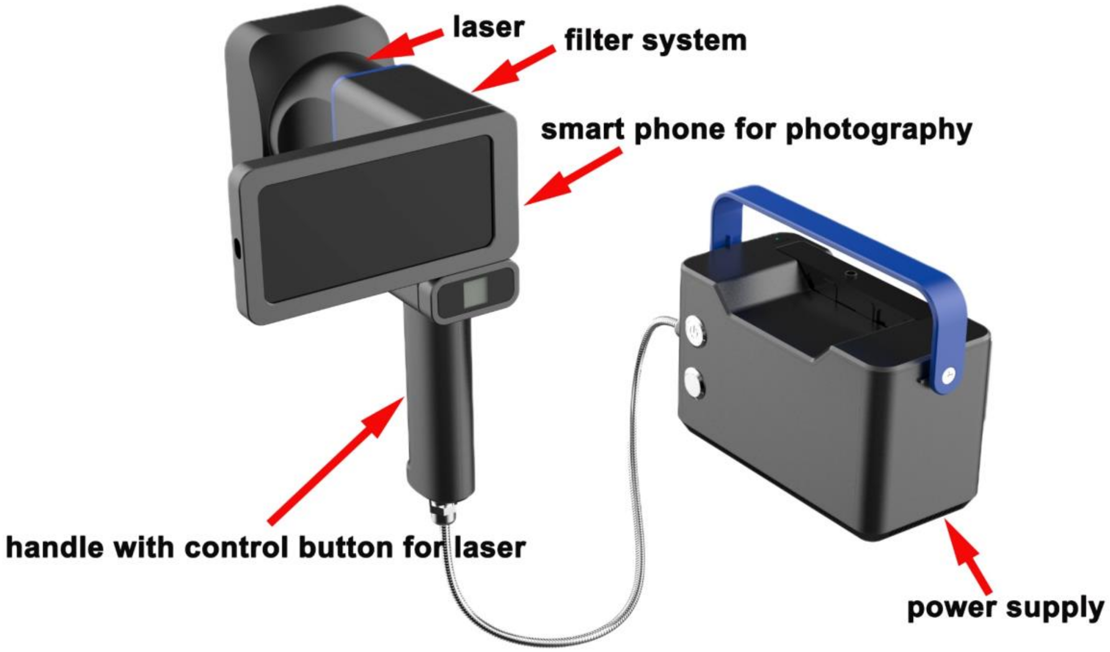

Figure S13. The portable photographic system for capturing LFPs. Power supply is a rechargeable lithium-ion battery purchased from SHENZHEN ZITAY TECH. CO. LTD. (Product model: CC-H75. Nominal Voltage: 10.8 V. Rated Capacity: 6800 mAh). The laser is semiconductor laser (λ = 445 ± 5 nm, 10 W) that was used for excitation. The smart phone is Huawei nova 5z smart phone with camera (pixel 2340 × 1080) used for photography and data storage. The optical low-pass filter is a light filter system that transmits wavelengths greater than 500 nm, with a cut-off rate of OD5.

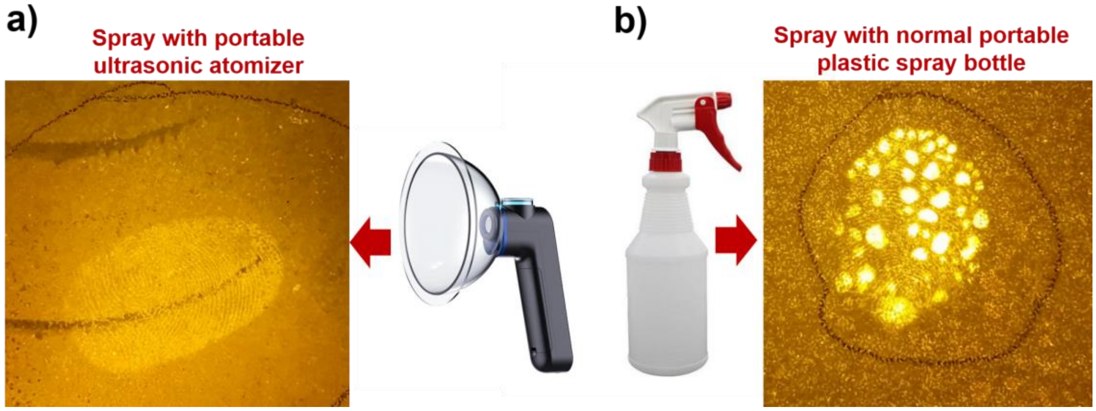

Figure S14. The ultrasonic atomizer ensures a homogenous and gentle spraying to cover the substrates. a) Spraying of a LFP-Red solution with portable ultrasonic atomizer for visualization of LFPs on wood board. b) Spraying of a LFP-Red solution with normal portable plastic spray bottle.

2.5 Grayscale plot for determining the optimal concentration of the probes and sensitivity of the probes

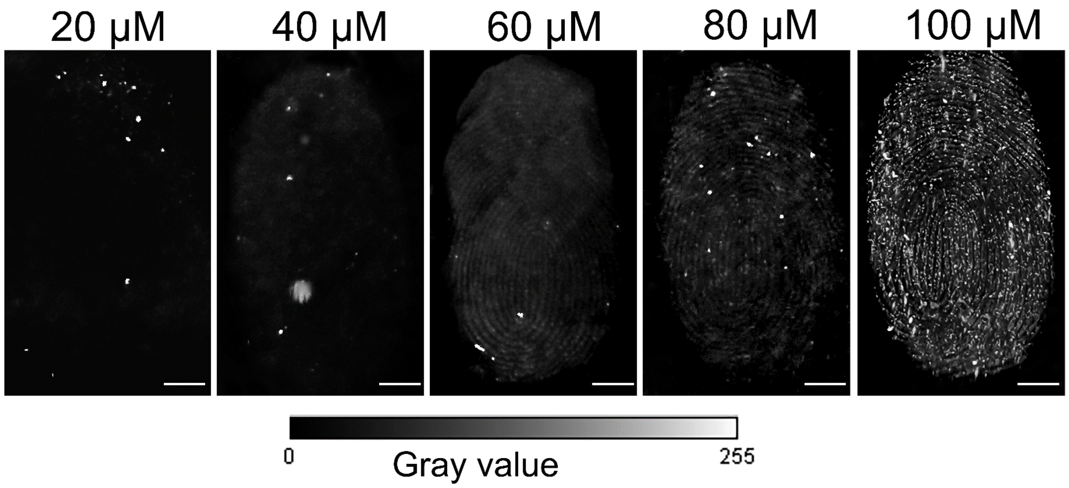

Figure S15. Grayscale images of LFPs on the tinfoil after spraying with different concentrations of LFP-Yellow solution for 10 seconds (scale bars: 5 mm, under 445 nm irradiation).

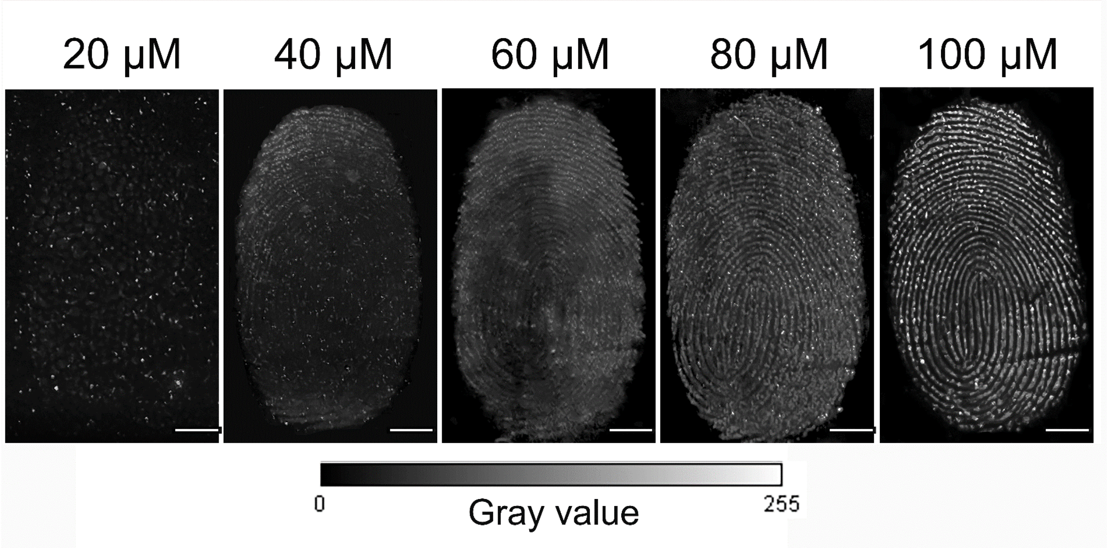

Figure S16. Grayscale images of LFPs on the acrylic plate after spraying with different concentrations of LFP-Red solution for 10 seconds (scale bars: 5 mm, under 445 nm irradiation).

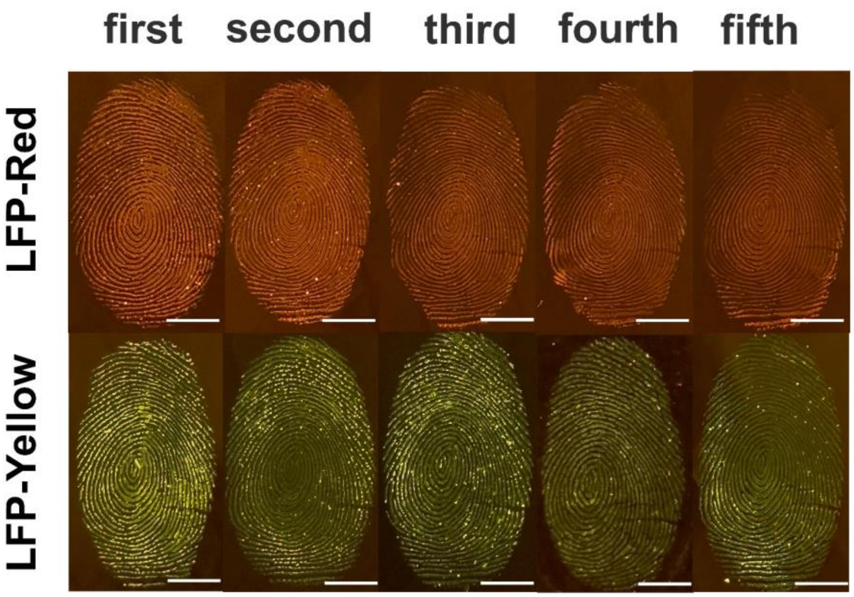

Figure S17. Actual color fluorescence photographs of LFPs taken after the same finger was repeatedly (5 times) placed in contact with tinfoil. The LFPs were developed by spraying with aqueous solutions of LFP-Red (100 μM) (a) and LFP-Yellow (100 μM), respectively. (Scale bars: 5 mm, under 445 nm irradiation).

2.6 Cytotoxicity assay for LFP-Red and LFP-Yellow

HeLa cells were grown in minimum Eagle's medium (MEM, high glucose) supplemented with 10% fetal bovine serum (FBS, GIBCO). Cells were incubated in 95% air and 5% CO_2 at 37 °C and typically passaged with a sub-cultivation ratio of 1:3 every 3 days. The cytotoxicity of LFP-Red and LFP-Yellow to HeLa cells was evaluated using the MTT assay. The concentration of LFP-Red and LFP-Yellow ranged from 70 to 110 μM, and the HeLa cells were incubated with LFP-Red and LFP-Yellow for 6 h, respectively.

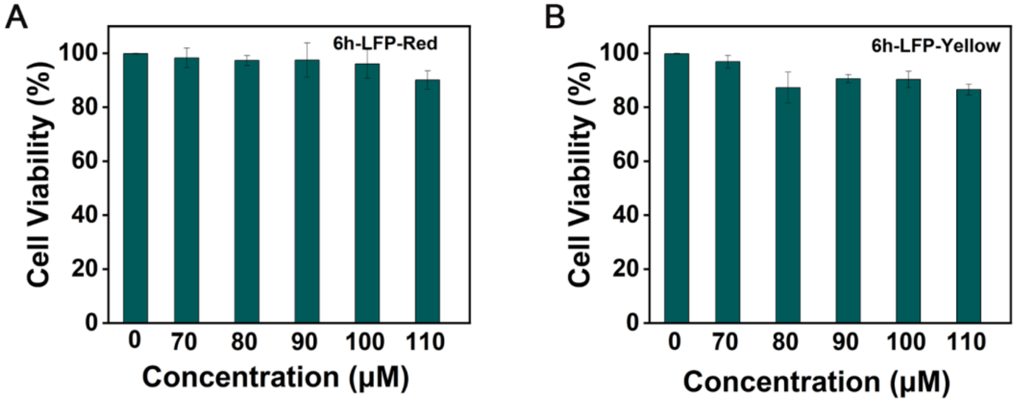

Figure S18. (A) Cell viability of HeLa cells stained with different concentrations of LFP-Red. (B) Cell viability of HeLa cells stained with different concentrations of LFP-Yellow. Error bars represent s.d..

The normal cells LO2 were grown in RPMI 1640 medium supplemented with 10% fetal bovine serum (FBS, GIBCO). Cells were incubated in 95% air and 5% CO_2 at 37 °C and typically passaged with a sub-cultivation ratio of 1:3 every 3 days. The cytotoxicity of LFP-Red and LFP-Yellow to LO2 cells was evaluated using the CCK-8 assay. The concentration of LFP-Red and LFP-Yellow ranged from 70 to 110 μM, and the LO2 cells were incubated with LFP-Red and LFP-Yellow for 6 h, respectively.

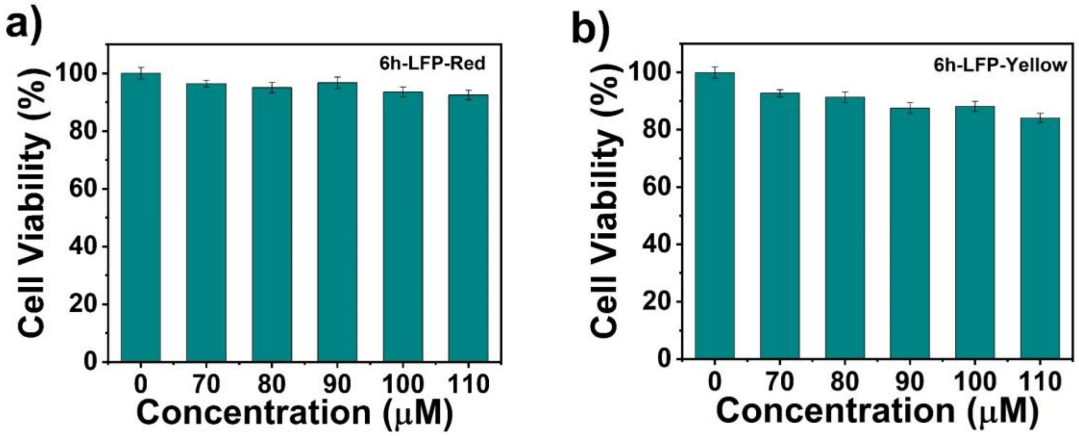

Figure S19. (A) Cell viability of LO2 cells stained with different concentrations of LFP-Red. (B) Cell viability of LO2 cells stained with different concentrations of LFP-Yellow. Error bars represent s.d..

2.7 Fluorescence performance of the control probes

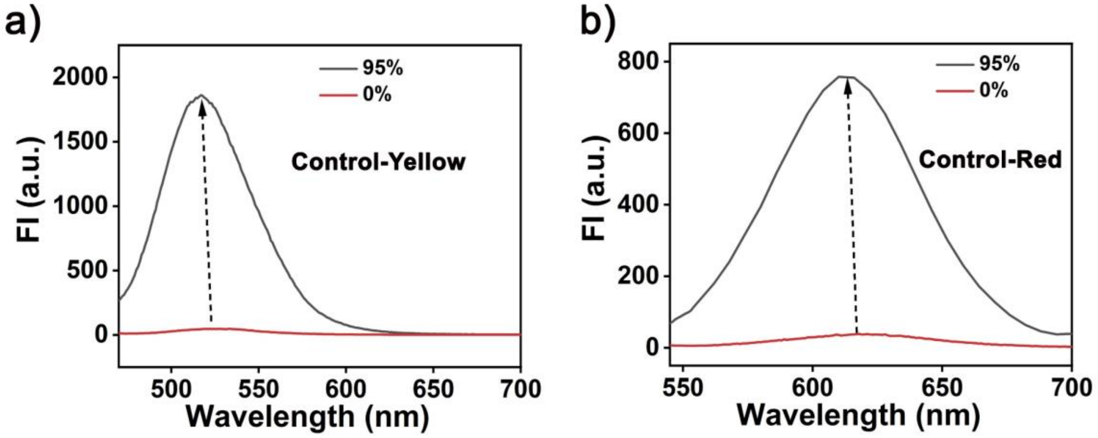

Figure S20. Fluorescence spectra of Control-Yellow (a) and Control-Red (b) in pure water (50 μM) with different fractions of glycerol (0% indicated pure water, and 95% indicated the 95 percent of glycerol in pure water). λ_{ex} = 453 nm, λ_{em} = 526 nm for Control-Yellow, λ_{ex} = 538 nm, λ_{em} = 623 nm for Control-Red. (Slit width ex = 10 nm and em = 10 nm; temperature 25°C).

2.8 Influence of fatty acids and DNA on LFP detection

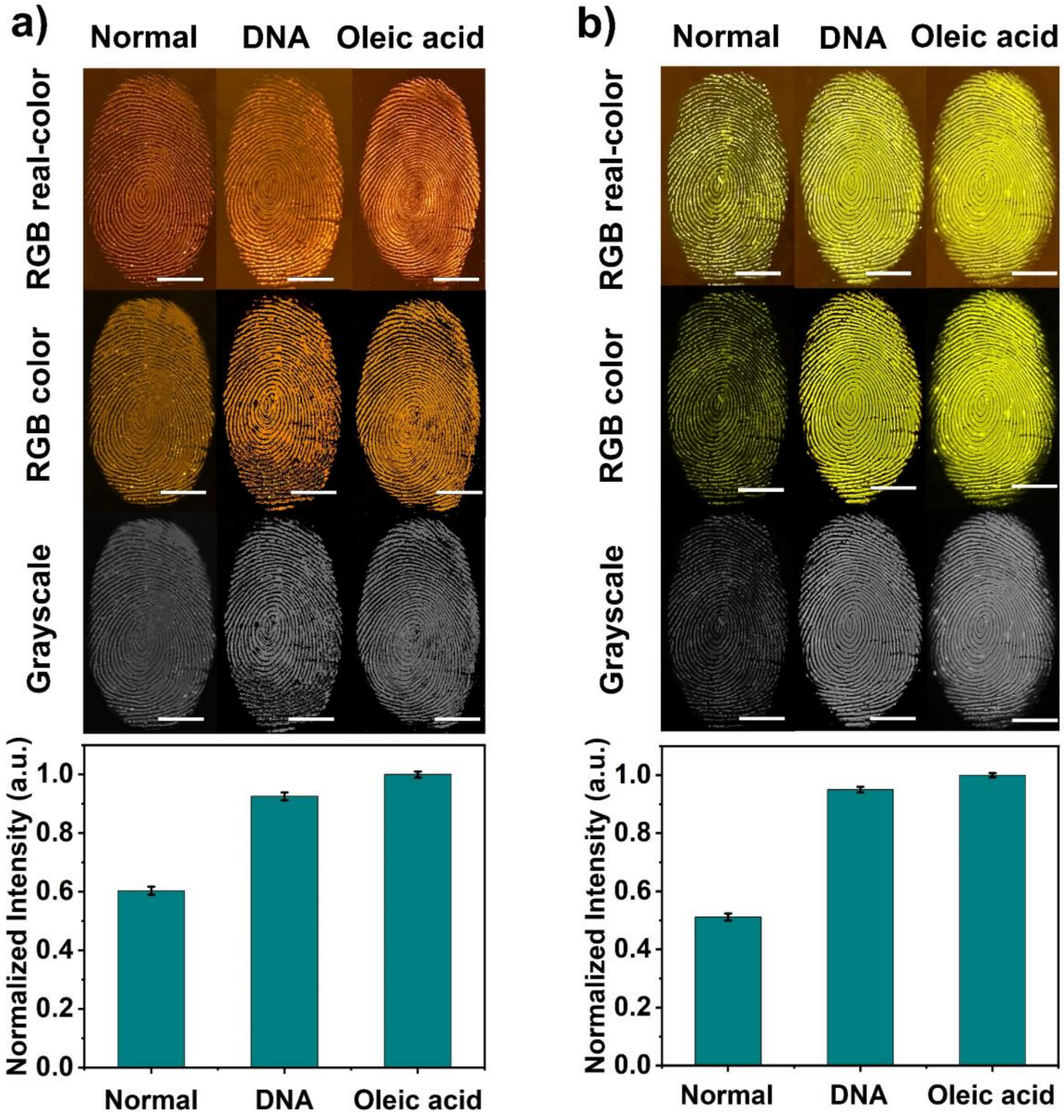

Figure S21. Top lane: Actual color fluorescence photographs, RGB color (pseudo color) fluorescence photographs and grayscale images of LFPs on tinfoil. The LFPs were formed by index finger in contact with the blank, DNA (salmon sperm, 1 mg/mL) and Oleic acid (100 μM), respectively. The LFPs were developed by spraying with the aqueous solution of LFP-Red (100 μM) (a) and LFP-Yellow (100 μM) (b) for 10 seconds, respectively. (Under 445 nm irradiation). Bottom lane: Semi-quantitative analysis of the fluorescence intensity of the images according to the top lanes with Image J software. Error bars represent three independent assays. (Scale bars: 5 mm, under 445 nm irradiation)

2.9. Comparison of LFP-Red and LFP-Yellow with 1,2-indanedione [3-4]

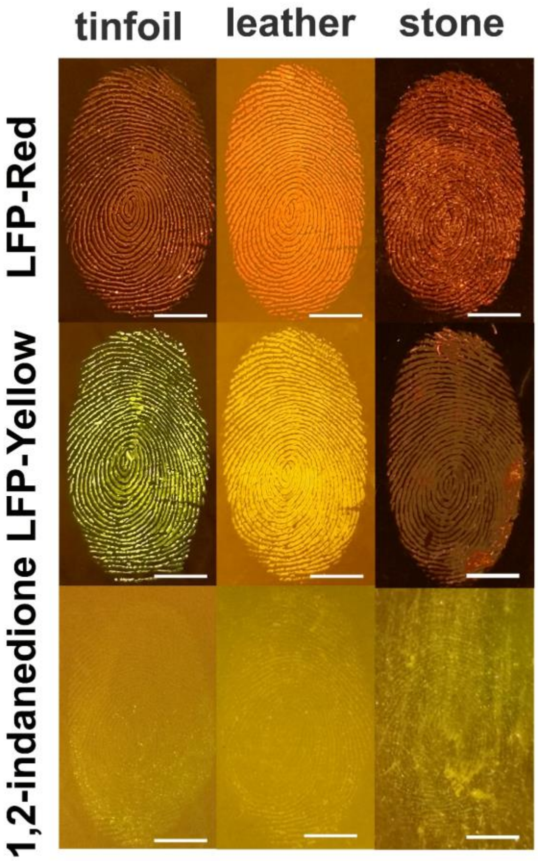

Figure S22. Actual color fluorescence photographs of LFPs on tinfoil, leather, and stone developed by spraying with the aqueous solution of LFP-Red, LFP-Yellow and 1,2-indanedione for 10 seconds, respectively. The concentration of LFP and LFP-Yellow is 100 μM. The solution of 1,2-indanedione was prepared according to the formula[3]: 0.2 g of 1,2-indanedione, 22.5 mL of ethyl acetate, 2.5 mL of glacial acetic acid, 225 mL of HFE-7100, 1 mL of $ZnCl_2$ solution. (Scale bars: 5 mm, LFP-Red and LFP-Yellow under 445 nm irradiation, 1,2-indanedione under 530 nm irradiation)

2.10 Scope of surfaces on which LFP can be detected

To further confirm the general nature of this method, more difficult surfaces of objects, such as the adhesive side of tapes, decorative surfaces of non-adsorbent objects, rough surfaces of semi-adsorbent stones, bricks and wood were used as substrates for evaluating the visualization of latent fingerprints.

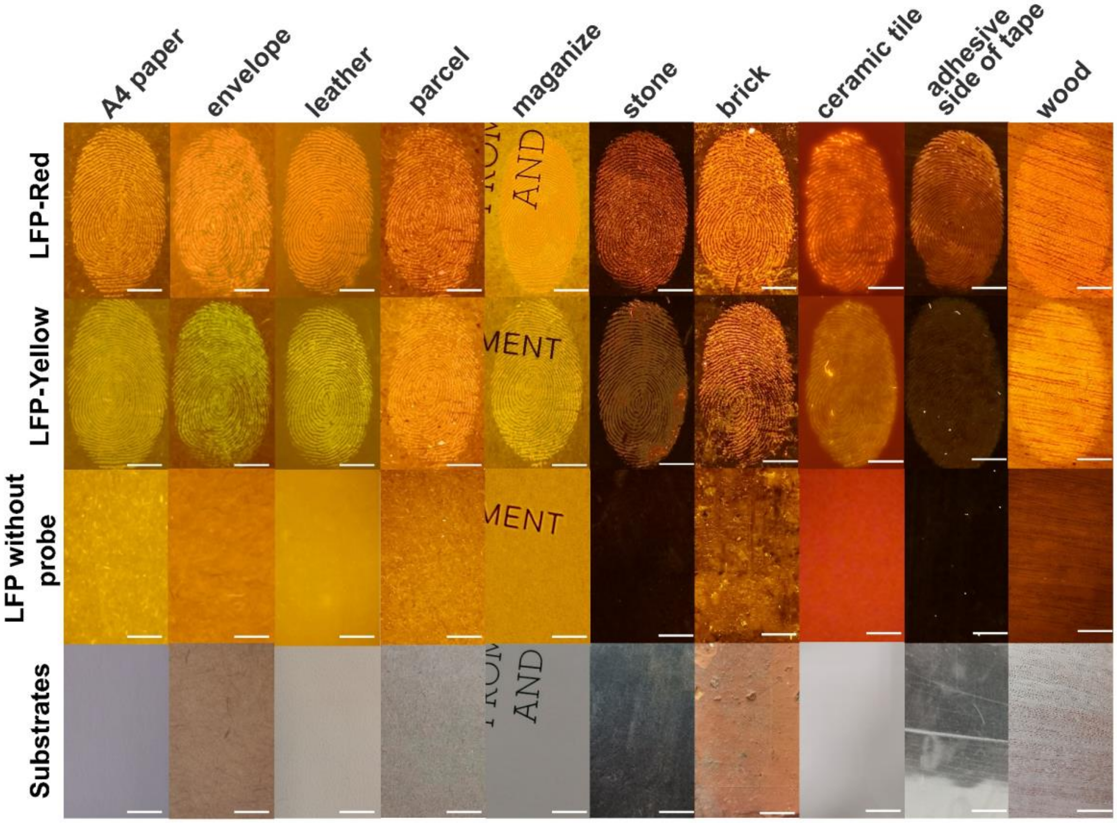

Figure S23. Actual color fluorescence photographs of LFPs on objects with more difficult surfaces developed by spraying with an aqueous solution of LFP-Red and LFP-Yellow, respectively. The images were taken using our photographic system. The concentration of the dyes is 100 µM. (Scale bars: 5 mm, under 445 nm irradiation).

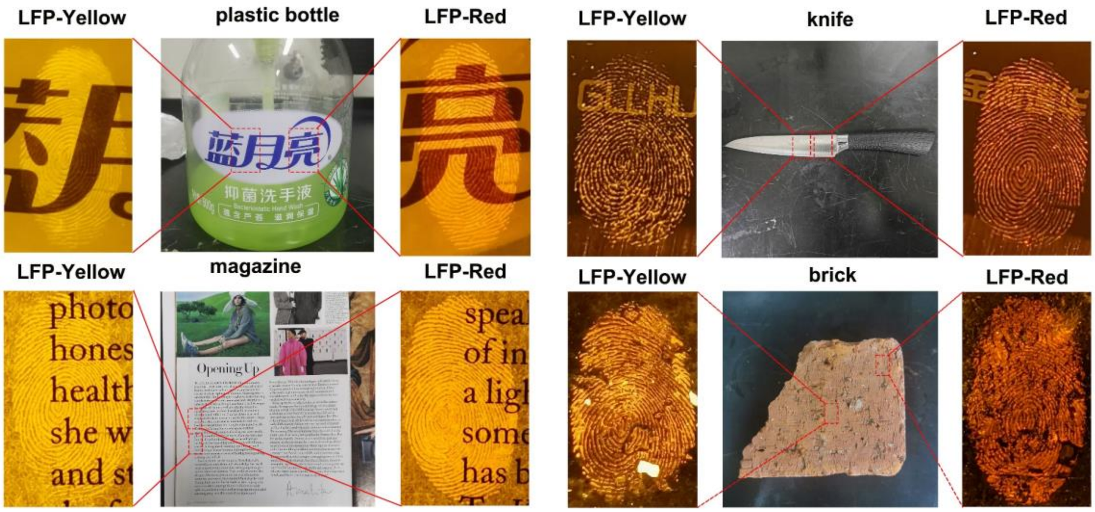

Figure S24. Actual color fluorescence photographs of natural latent fingerprints. Latent fingerprints developed by LFP-Red and LFP-Yellow, respectively. Using a plastic bottle, knife, magazine and brick. The visualized latent fingerprint images can be observed in the enlarged areas of the surface of these objects. The latent fingerprints were formed by contact of the same finger directly (the finger was not washed or additionally cleaned before forming the latent fingerprints) with the surface of the substrates including a plastic bottle, knife, magazine, and brick.

2.11 RGB and grayscale photographs of LFPs

a. An aqueous solution of the probe (100 μM) was added to the portable ultrasonic atomizer and sprayed on the surface of substrates with fingerprints (ceramics, steel, plastic, glass, tinfoil, acrylic plate) for 10 seconds. Without any post-treatment, images of developed LFPs were acquired with the portable photographic system under 445 nm excitation.

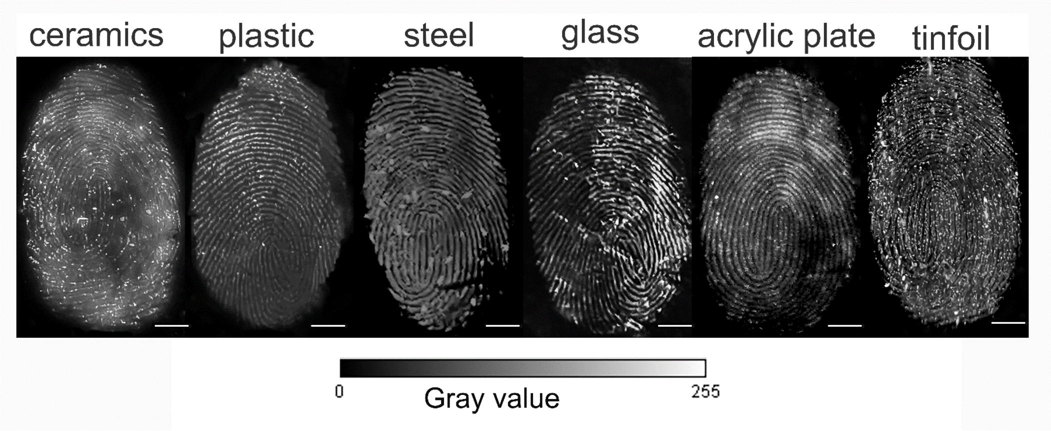

Figure S25. Grayscale images of LFPs on different substrates developed by spraying with an aqueous solution of LFP-Yellow (scale bars: 5 mm, under 445 nm irradiation).

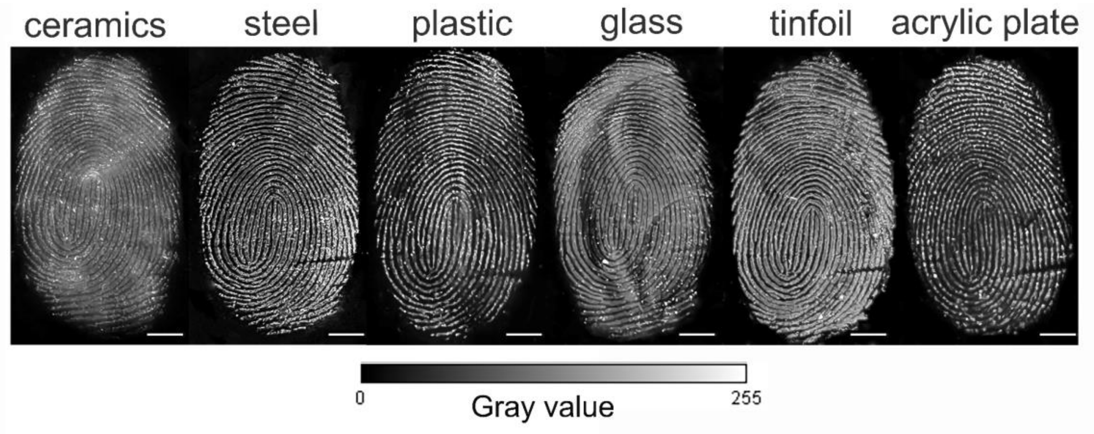

Figure S26. Grayscale images of LFPs on different substrates developed by spraying with an aqueous solution of LFP-Red (scale bars: 5 mm, under 445 nm irradiation).

b. We zoomed in on the fingerprint photo (Figure 4, Figure S27, Figure S28, Figure S29). Clear and consistent LFPs images with Level 2 and Level 3 details were observed.

According to the literature, fingerprint patterns can be classified into 3 levels. Level 1 is the global level that refers to the overall pattern formed by the flow of papillary ridges on the papillary surface. Traditionally, the general pattern formed on the fingertips has been classified into generic classes. Level 2 is at the local level that refers to major ridge path deviations, also known as minutiae, points of identification, or Galton characteristics. The two most prominent ridge characteristics, called minutiae are ridge endings and ridge bifurcations. Level 3 is at the very fine level that refers to

intrinsic or innate ridge formations: the alignment and shape of each ridge unit, pore shape, and relative pore positions.[5-6]

Analyzing levels 1-3 of fingerprints involves examining and comparing the ridge patterns, minutiae, and overall characteristics of the fingerprints. Here is a general overview of the analysis process. Level 1: Ridge flow and pattern. Ridge flow is the determination of the overall direction of ridge flow in the fingerprint, which could be classified into different patterns. And there are three principal pattern types including the loops, whorls, and arches[7]. Level 2: Minutiae Points. Identify and count the minutiae points, which are the ridge characteristics. Common types include ridge endings, bifurcations, and short ridges. These points help establish the uniqueness of a fingerprint. Minutiae Comparison: Comparing the minutiae points between different fingerprints to determine if they match or differ. This step is crucial in fingerprint identification. Level 3: Overall Characteristics. Ridge Count: Count the number of ridges between specific points, such as the core or delta, to establish consistency and uniqueness. Ridge Quality: Assess the clarity and continuity of the ridge patterns.

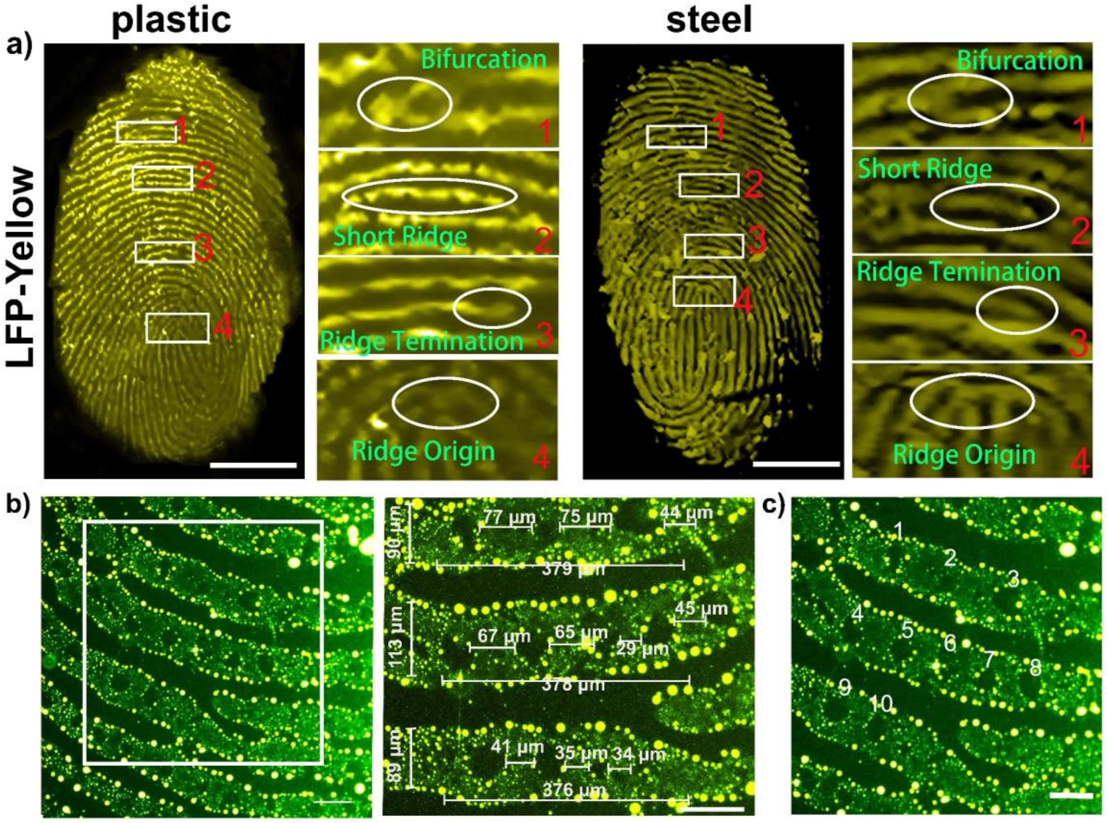

Figure S27. Level 1-3 details of LFP-Yellow (100 μM) treated LFPs. (a) Level 1 and Level 2 details of LFPs on plastic and steel were clearly visualized using RGB color (pseudo color) fluorescence images by enlarging the partial regions of LFPs. Ovals in 1, 2, 3, 4 insets spotlight the bifurcation, ridge origin, ridge termination, and short ridge, respectively. (Scale bars: 5 mm, under 445 nm irradiation). (b, c) The level 3 detail was visualized by fluorescence microscopic images for the partial region of LFPs. Scale bar: 100 μm.

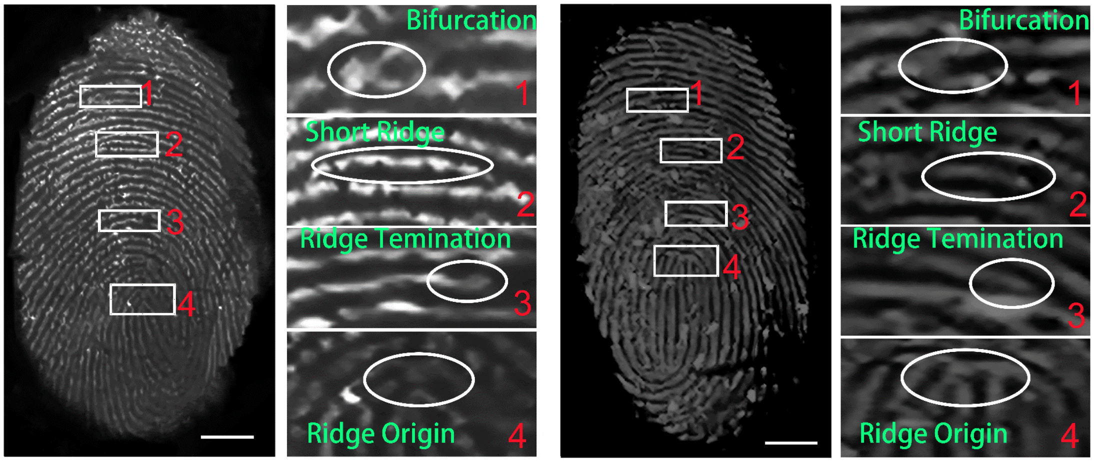

Figure S28. Level 2 details grayscale images of LFPs on plastic (left) and steel (right) developed with LFP-Yellow aqueous solution (100 μM) under 445 nm irradiation. (Scale bars: 5 mm).

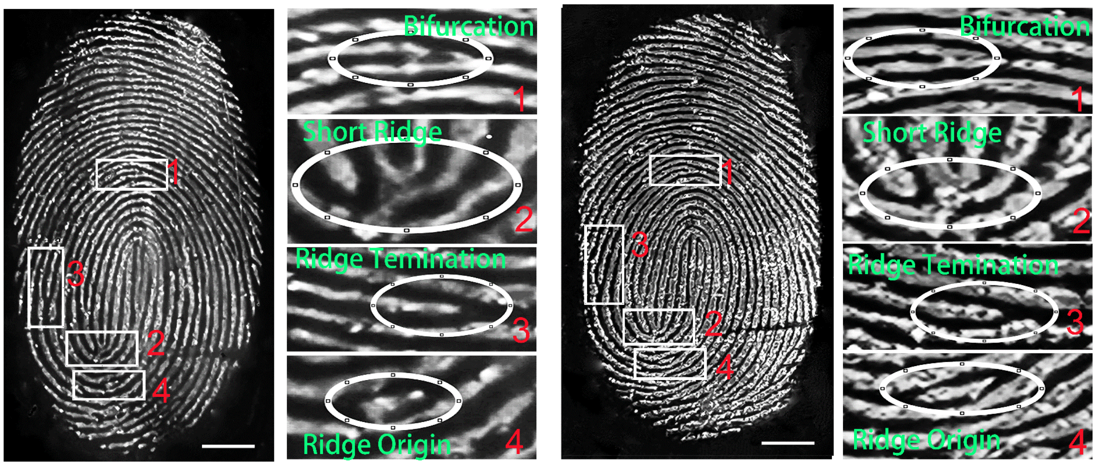

Figure S29. Level 2 details grayscale images of LFPs on plastic (left) and steel (right) developed with LFP-Red aqueous solution (100 μM) under 445 nm irradiation. (Scale bars: 5 mm).

c. Stability of the LFP-Red and LFP-Yellow

Figure 5(a), Figure S30(a), Figure S31(a), Figure S32(a): On three identical plastics, the same volunteer used the same finger to print three times respectively to produce three fingerprints, which were kept for 1, 4 and 7 days respectively, and then sprayed to obtain the fingerprint image.

Figure 5(c), Figure S30(c), Figure S31(c), Figure S32(c): Spray the existing fingerprint on plastic, store the probe for 2 months, and spray the fingerprint on plastic (with the same volunteer, newly printed fingerprint) to obtain the fingerprint image.

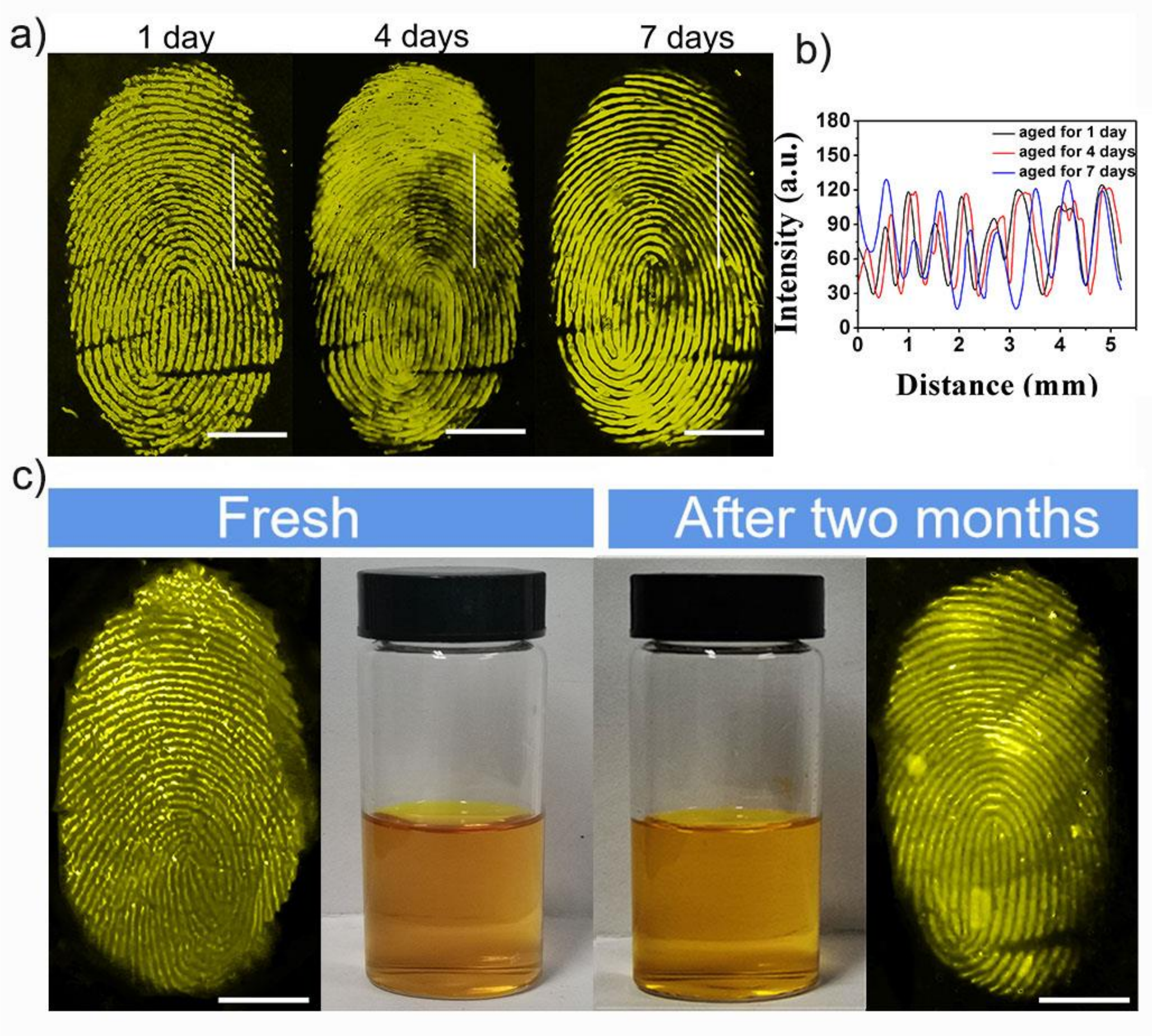

Figure S30. (a) RGB color (pseudo color) fluorescence photographs LFPs aged for 1 day, 4 days and 7 days on tinfoil are developed by LFP-Yellow (100 μM) aqueous solution. (b) Variations of the fluorescence intensity between the fingerprint ridge and furrow across the white line. (c) Fresh LFP-Yellow aqueous solution (100 μM) and after storing for 2 months was used for LFPs development on tinfoil using the spray method (the LFPs are from the same finger). (Scale bars: 5 mm, under 445 nm irradiation).

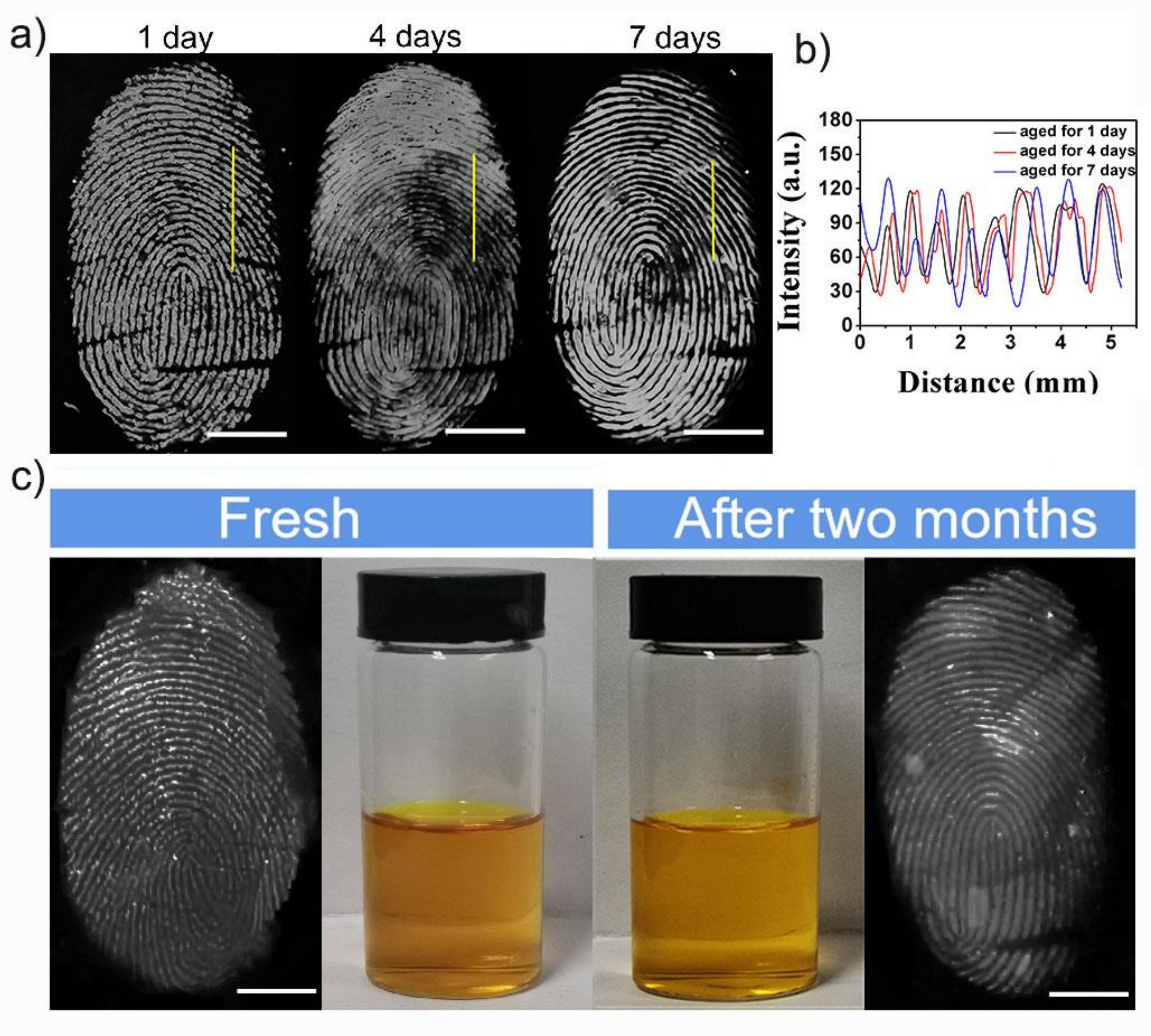

Figure S31. The grayscale images are corresponding to the RGB true color images of **Figure S30**. (a) LFPs aged for 1 day, 4 days and 7 days on tinfoil are developed by LFP-Yellow (100 μM) aqueous solution. (b) Variations of the fluorescence intensity between the fingerprint ridge and furrow across the red line. (c) Fresh LFP-Yellow aqueous solution (100 μM) and after storing for 2 months was used for LFPs development on tinfoil using the spray method. (Scale bars: 5 mm, under 445 nm irradiation).

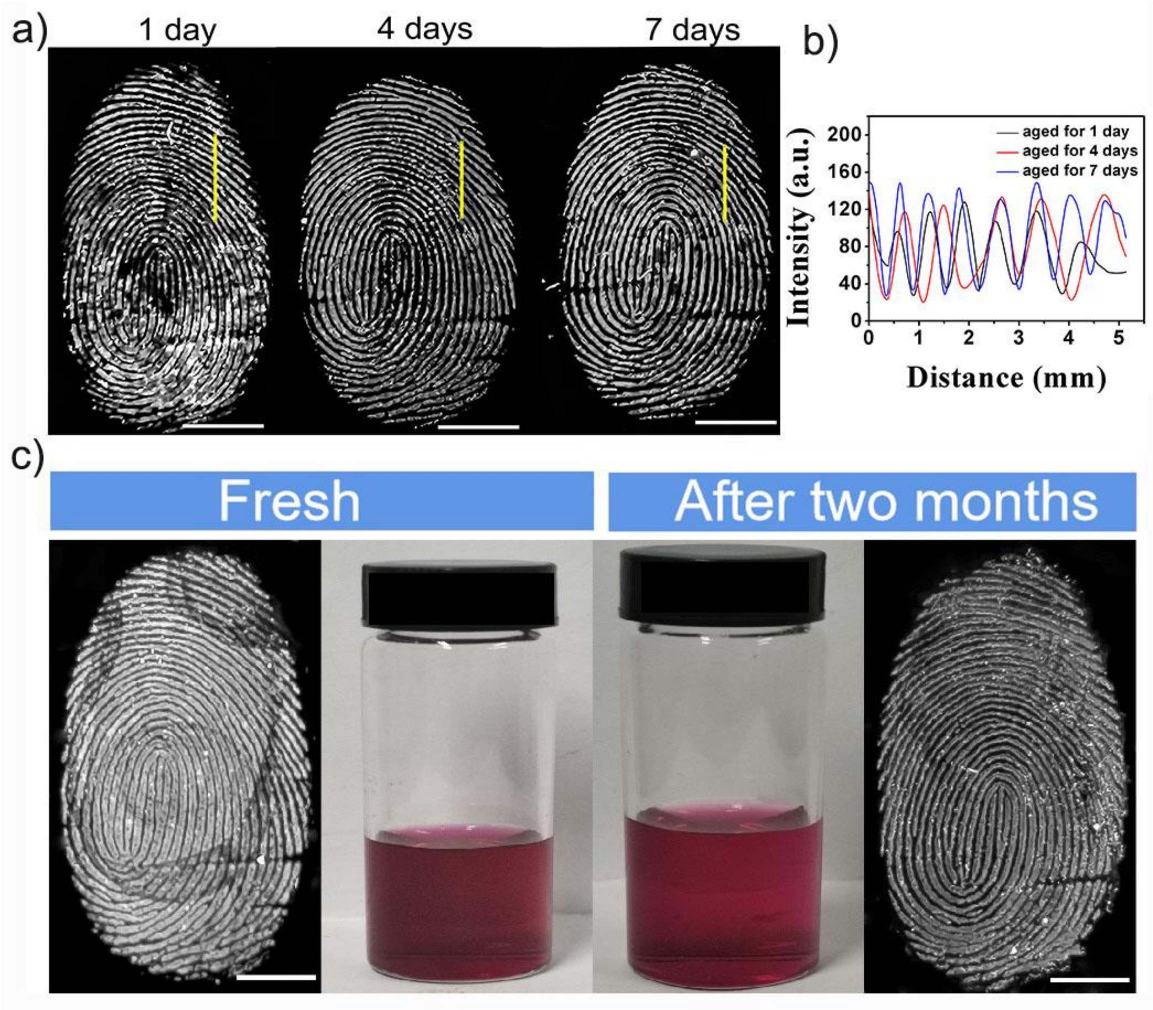

Figure S32. The grayscale images are corresponding to the RGB true color images of **Figure 5**. (a) LFPs aged for 1 day, 4 days and 7 days on tinfoil are developed by LFP-Red (100 μM) aqueous solution. (b)Variations of the fluorescence intensity between the fingerprint ridge and furrow across the yellow line. (c) Fresh LFP-Red aqueous solution (100 μM) and after storing for 2 months was used for LFPs development on tinfoil using the spray method (the LFPs are from the same finger). (Scale bars: 5 mm, under 445 nm irradiation).

2.12 Evaluation of the effect of LFP-Red and LFP-Yellow on the identification of DNA

STR analysis was conducted by using the MagicMag Micro Genomic DNA Extraction Kit. This assay was done by Changchun City Bokun Bio-Tech Co. Ltd. First, 50 μL of LFP-Red or LFP-Yellow solution was mixed with 50 μL diluted blood samples, respectively. Then 10 μL of probe solution was added dropwise to a clean blood sample collection card and dried to present the blood stains. Then a double swab technique[8] was used to collect the sample from the blood stains for DNA analysis. And the STR results were analyzed in Figure S33.

STR analysis of the DNA directly extracted from latent fingerprints or latent blood fingerprints was conducted by using the AGCU X19 STR kit (AGCU ScienTech Incorporation,Wuxi, Jiangsu, China). This assay was done by Guangzhou Forensic Science Institute, Guangzhou, China. The results are shown in Figure S34-S36.

The results of STR gene typing are usually presented in a numerical form, which represents the length of different STR sequences, with each number corresponding to one STR locus. For example, a short sequence named "D5S818" (Figure 5 or Figure S33) produces consistent results of "10, 12" before and after the probe spraying, we can conclude that the probe solution has no impact on the subsequent DNA extraction.

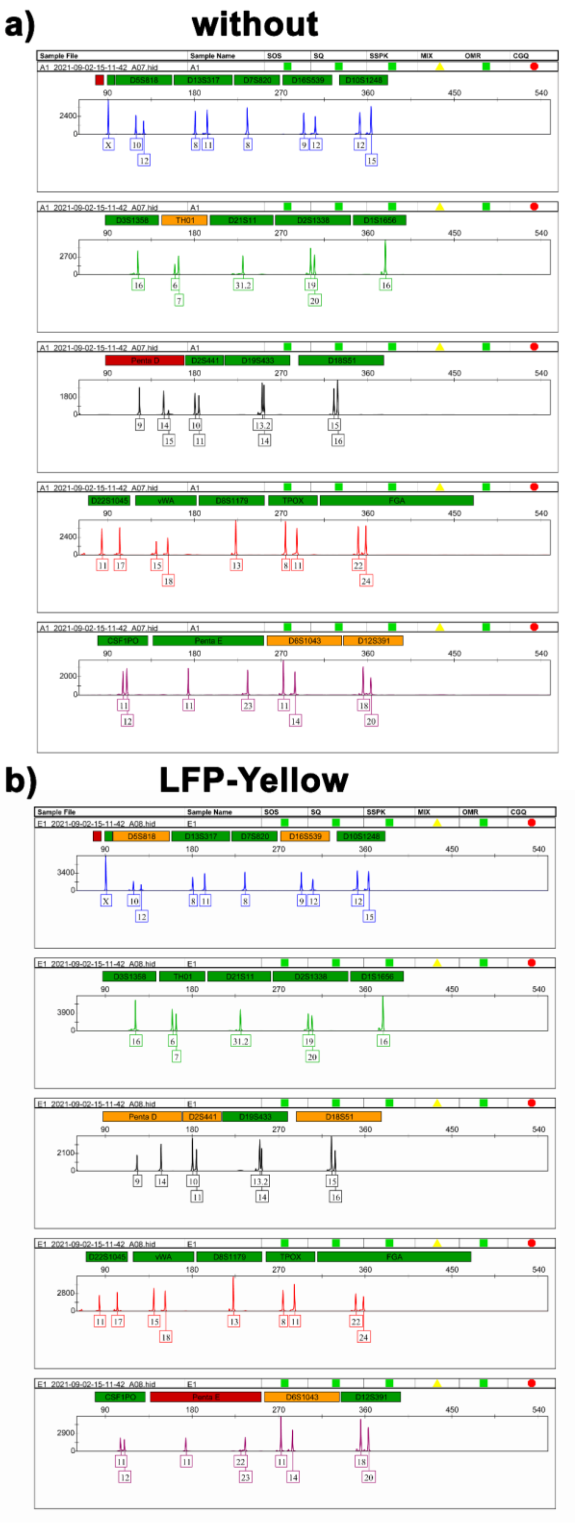

Figure S33. (a) STR analysis of DNA in blood samples. (b) STR analysis of DNA from blood stains after treatment with LFP-Yellow solution. (Green: extremely strong genetic locus signal intensity. Orange: strong genetic locus signal intensity. Red: moderate genetic locus signal intensity.)

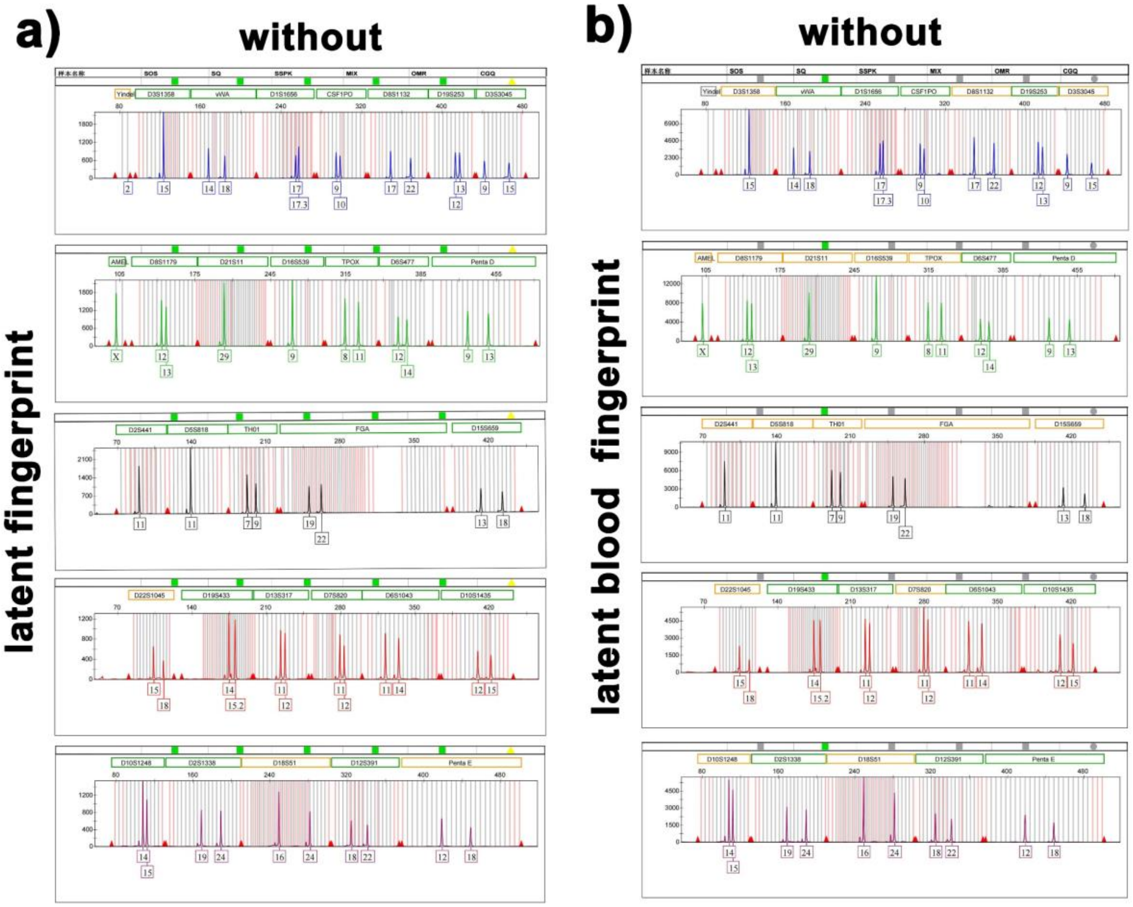

Figure S34. (a) STR analysis of DNA extracted directly from latent fingerprints. (b) STR analysis of DNA extracted directly from latent blood fingerprints. Latent blood fingerprints were formed by the index finger in contact with a trace sample of blood. (Green: extremely strong genetic locus signal intensity. Orange: strong genetic locus signal intensity. Red: moderate genetic locus signal intensity).

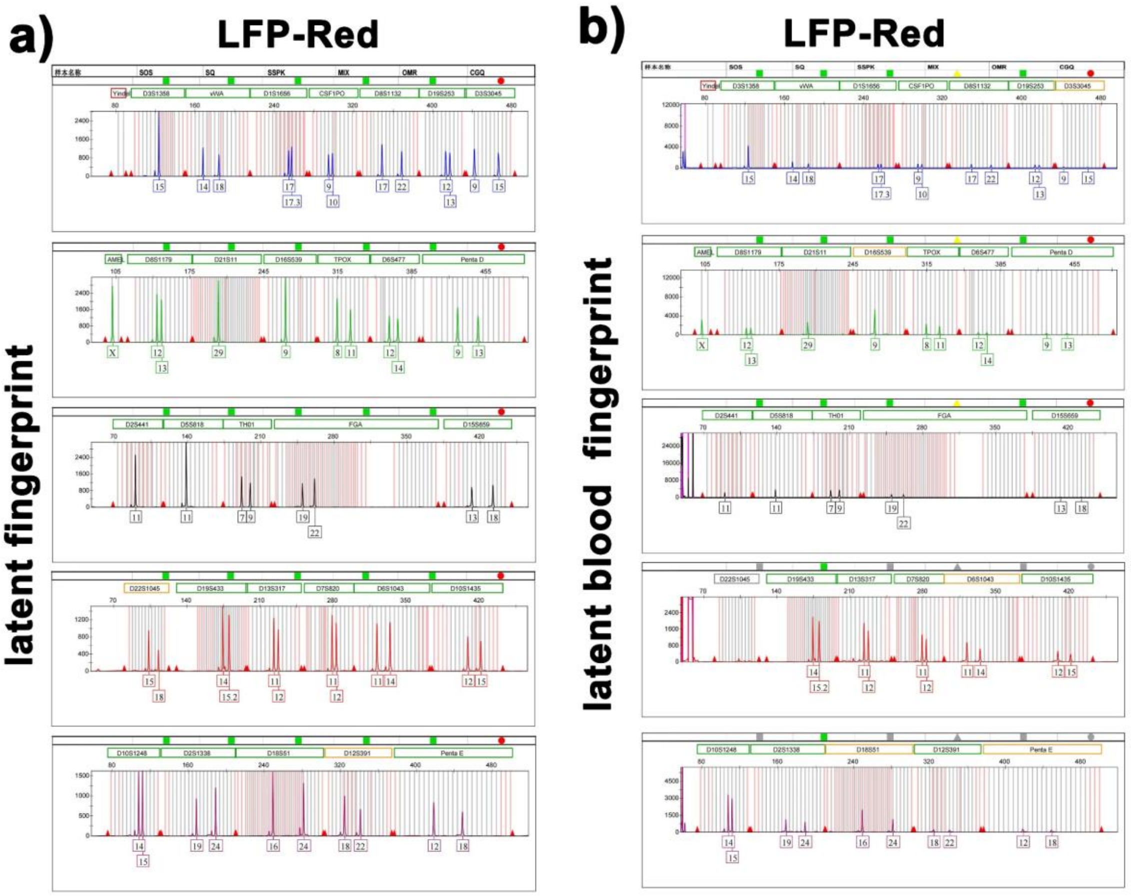

Figure S35. (a) STR analysis of DNA extracted directly from latent fingerprints after treatment with LFP-Red solution (100 μM). (b) STR analysis of DNA extracted directly from the latent blood fingerprints after treatment with LFP-Red solution (100 μM). Latent blood fingerprints were formed by the index finger in contact with a trace sample of blood. (Green: extremely strong genetic locus signal intensity. Orange: strong genetic locus signal intensity. Red: moderate genetic locus signal intensity).

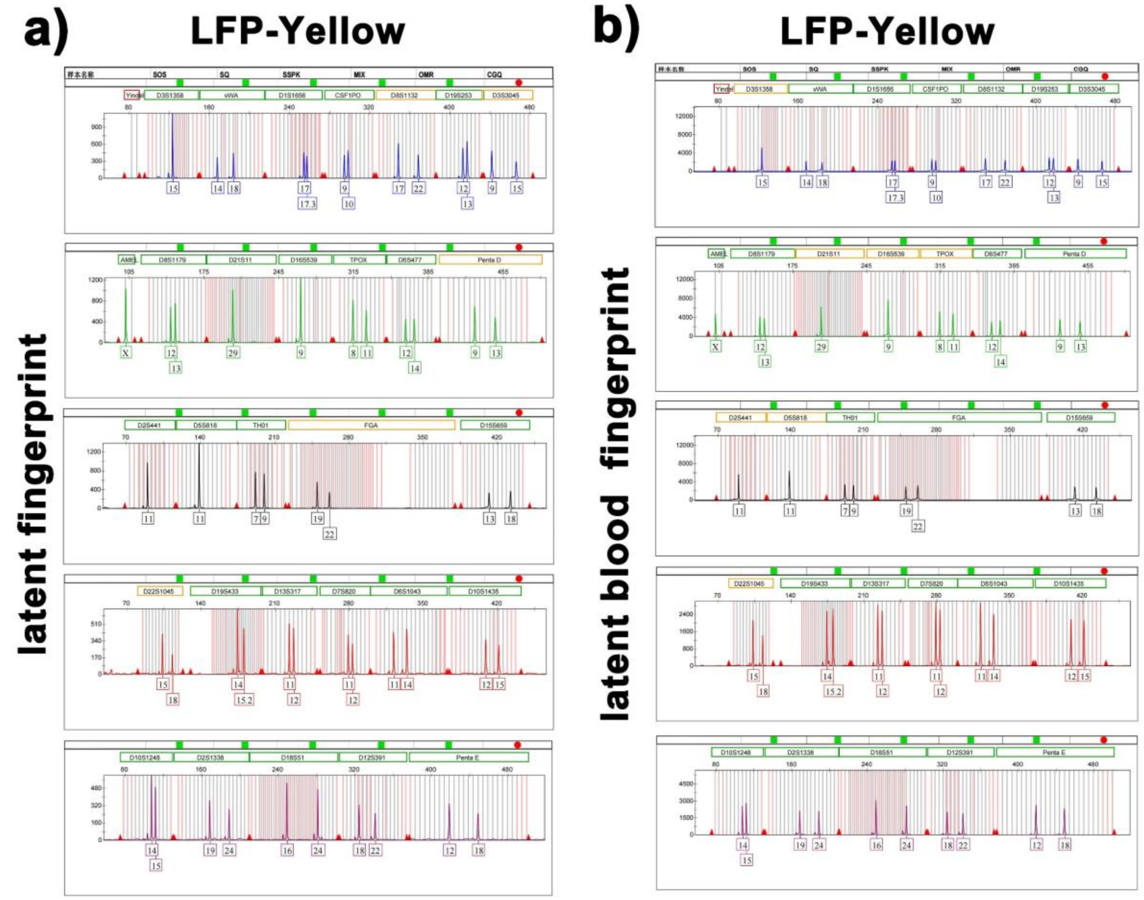

Figure S36. (a) STR analysis of DNA extracted directly from latent fingerprints after treatment with LFP-Yellow solution (100 μM). (b) STR analysis of DNA extracted directly from latent blood fingerprints after treatment with LFP-Yellow solution (100 μM). Latent blood fingerprints formed by the index finger in contact with a trace sample of blood. (Green: extremely strong genetic locus signal intensity. Orange: strong genetic locus signal intensity. Red: moderate genetic locus signal intensity).

3. NMR Spectra and HRMS

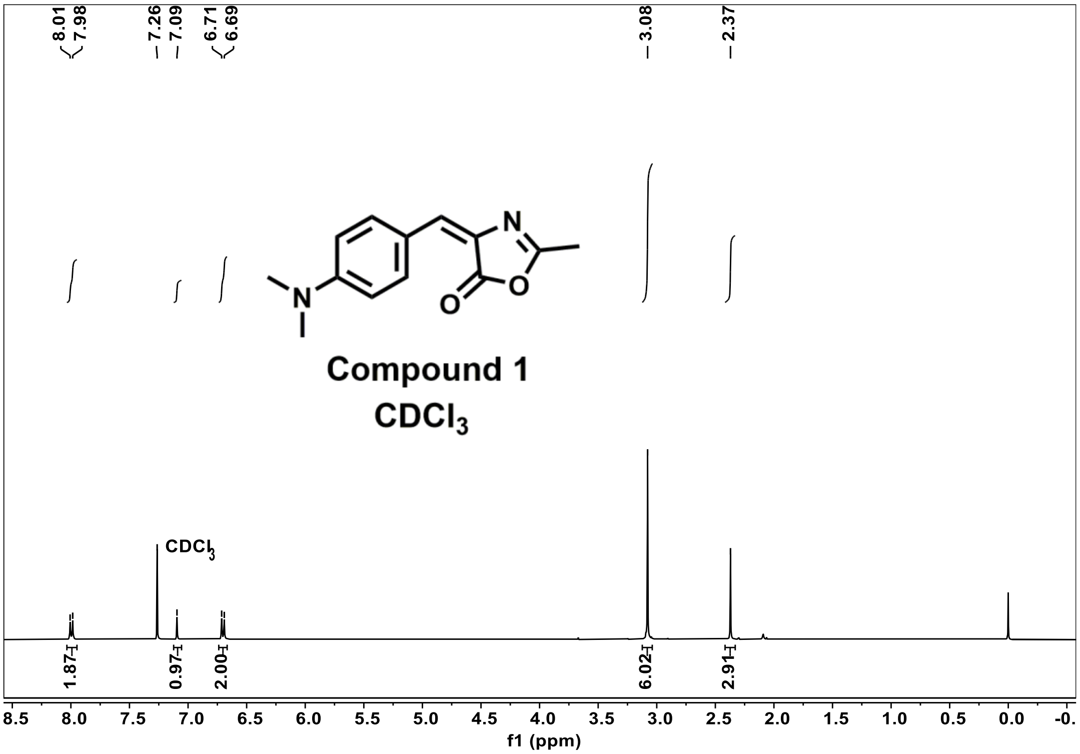

Figure S37. ^{1}H NMR spectrum of compound **1**.

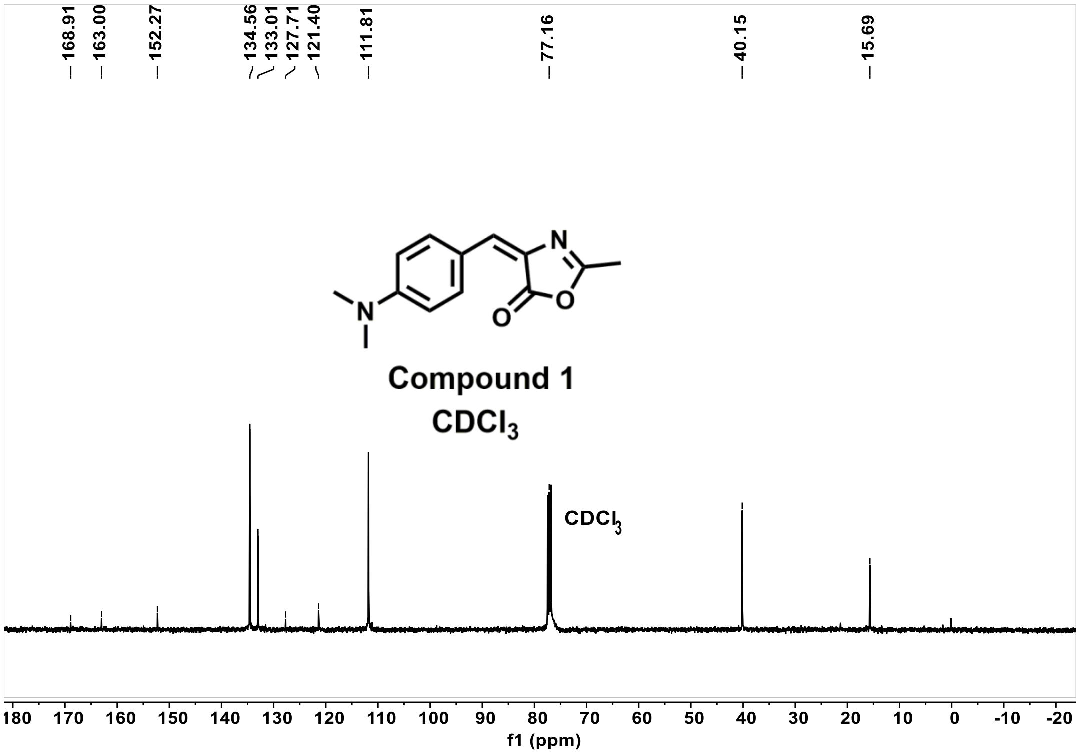

Figure S38. ^{13}C NMR spectrum of compound **1**.

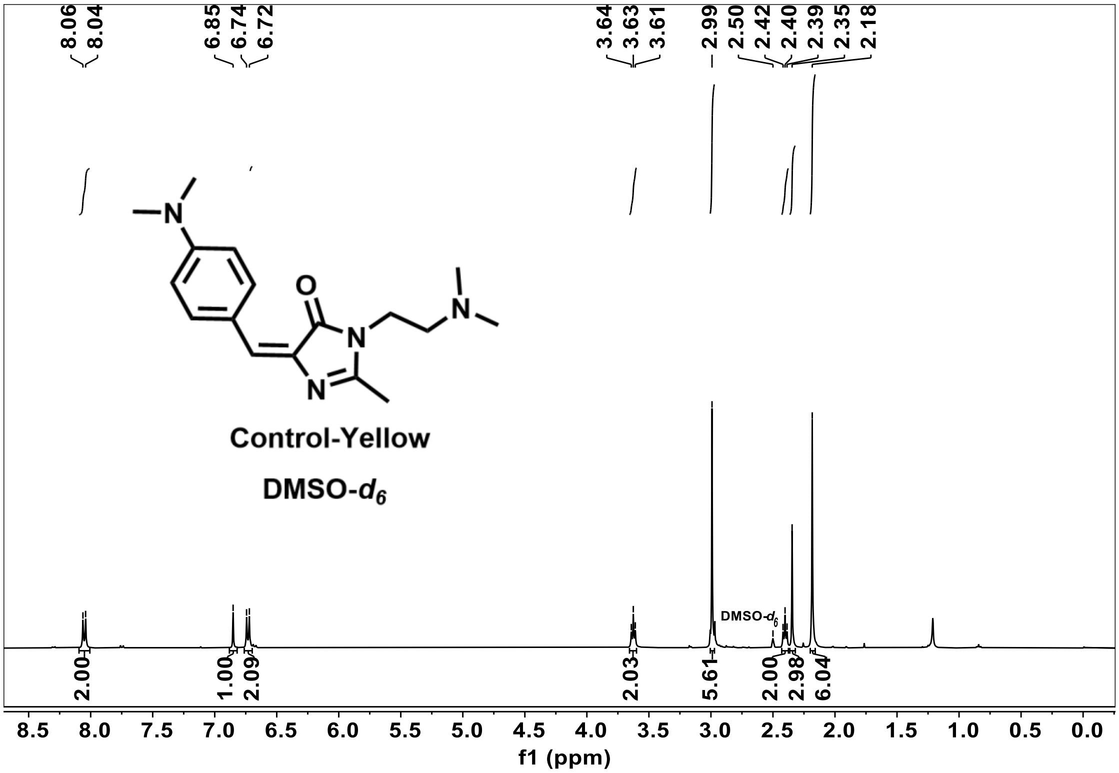

Figure S39. ^{1}H NMR spectrum of Control-Yellow.

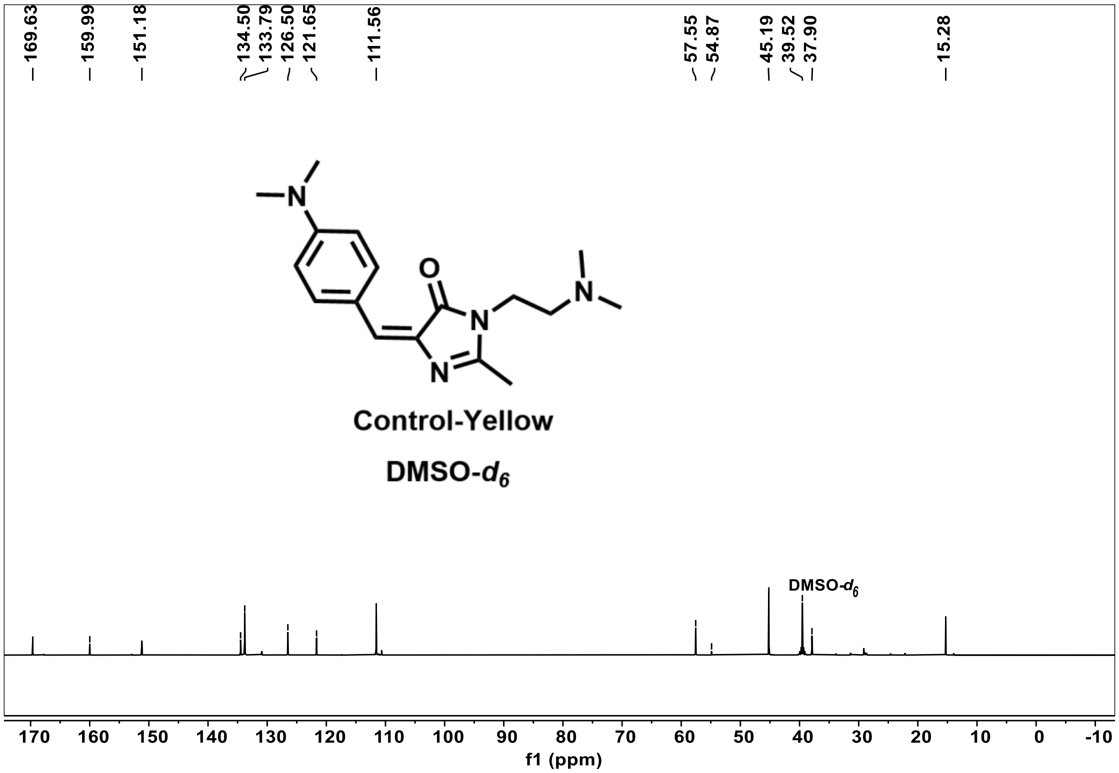

Figure S40. ^{13}C NMR spectrum of Control-Yellow.

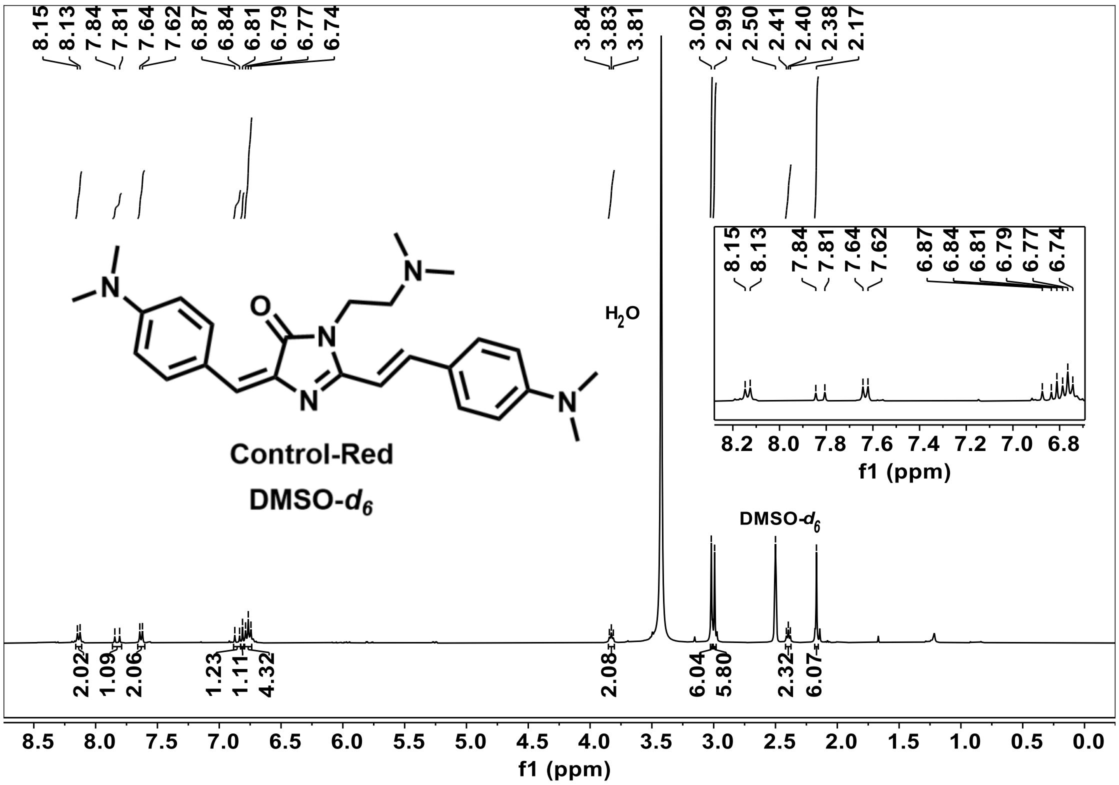

Figure S41. ^{1}H NMR spectrum of Control-Red.

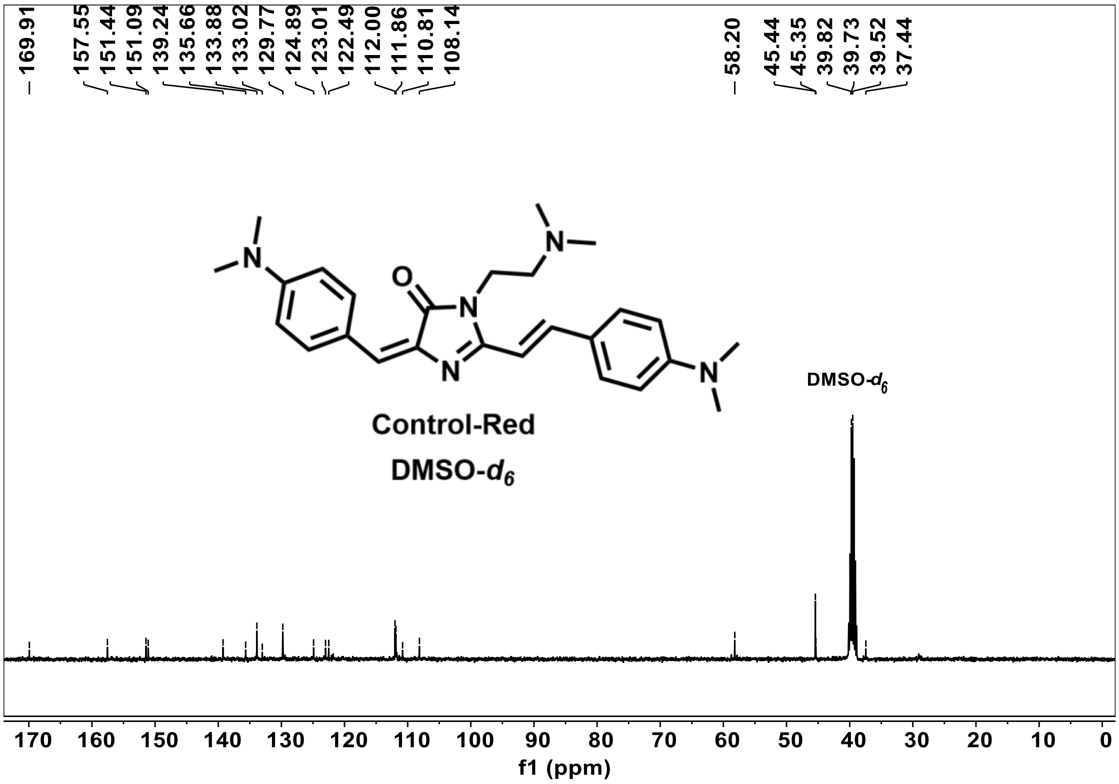

Figure S42. ^{13}C NMR spectrum of Control-Red.

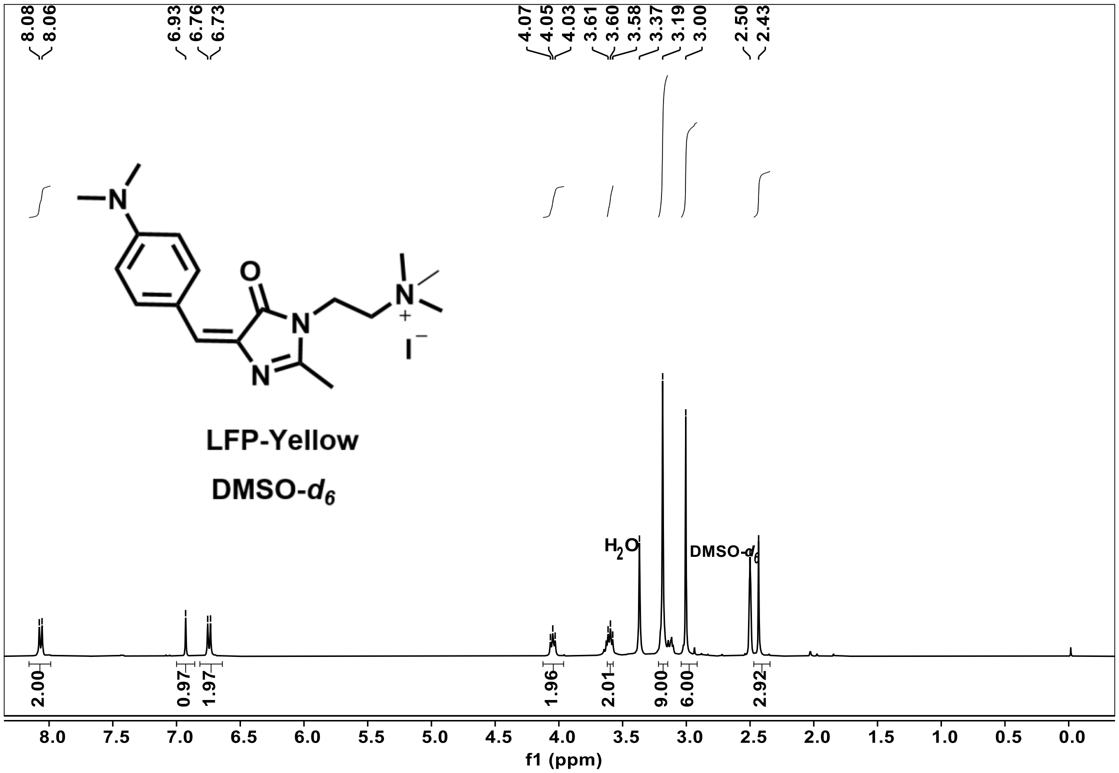

Figure S43. ^{1}H NMR spectrum of LFP-Yellow.

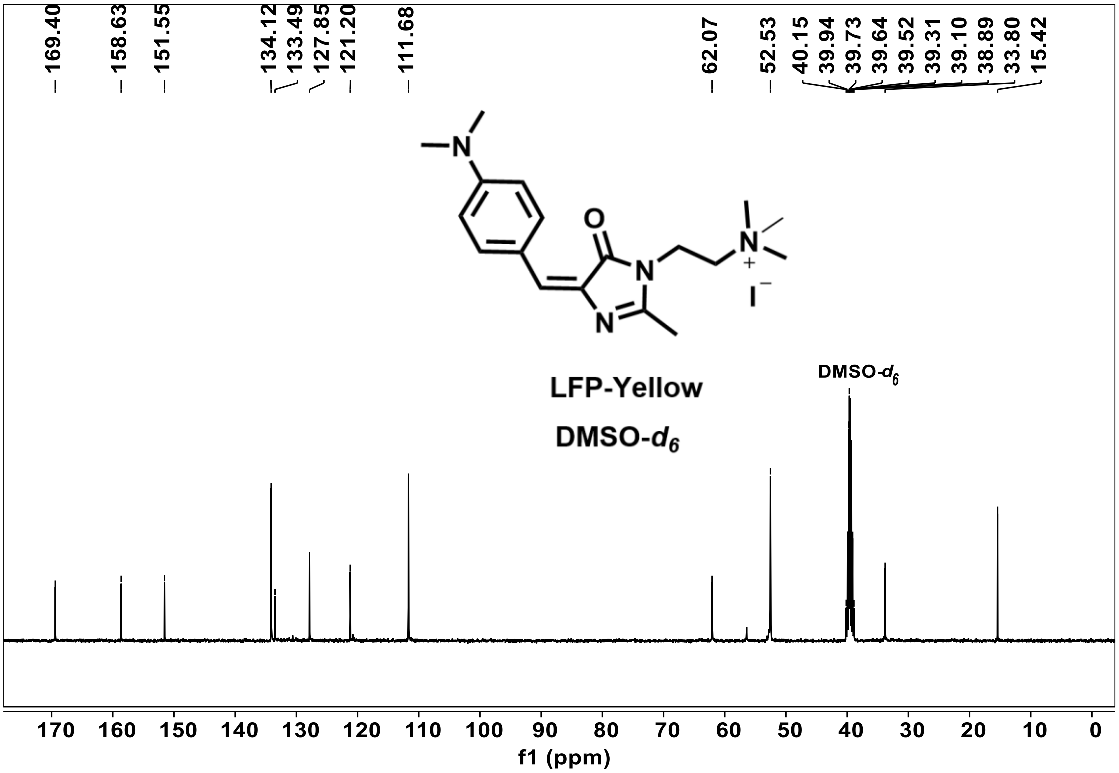

Figure S44. ^{13}C NMR spectrum of LFP-Yellow.

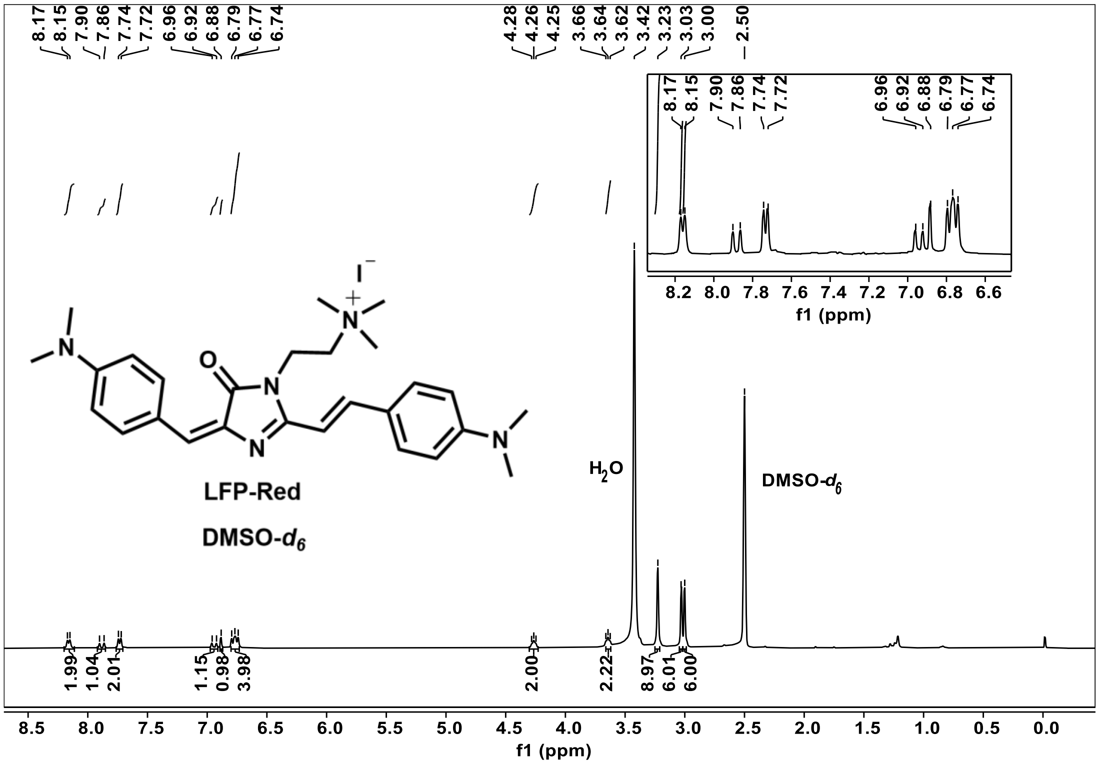

Figure S45. ^{1}H NMR spectrum of LFP-Red.

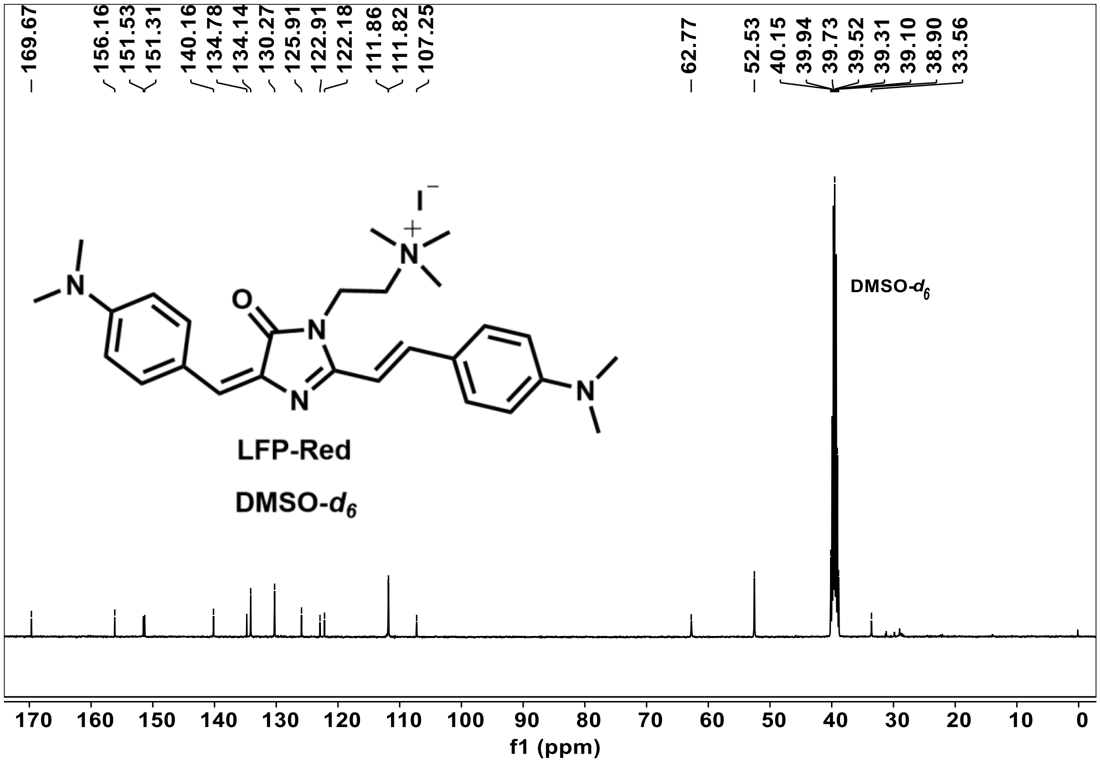

Figure S46. ^{13}C NMR spectrum of LFP-Red.

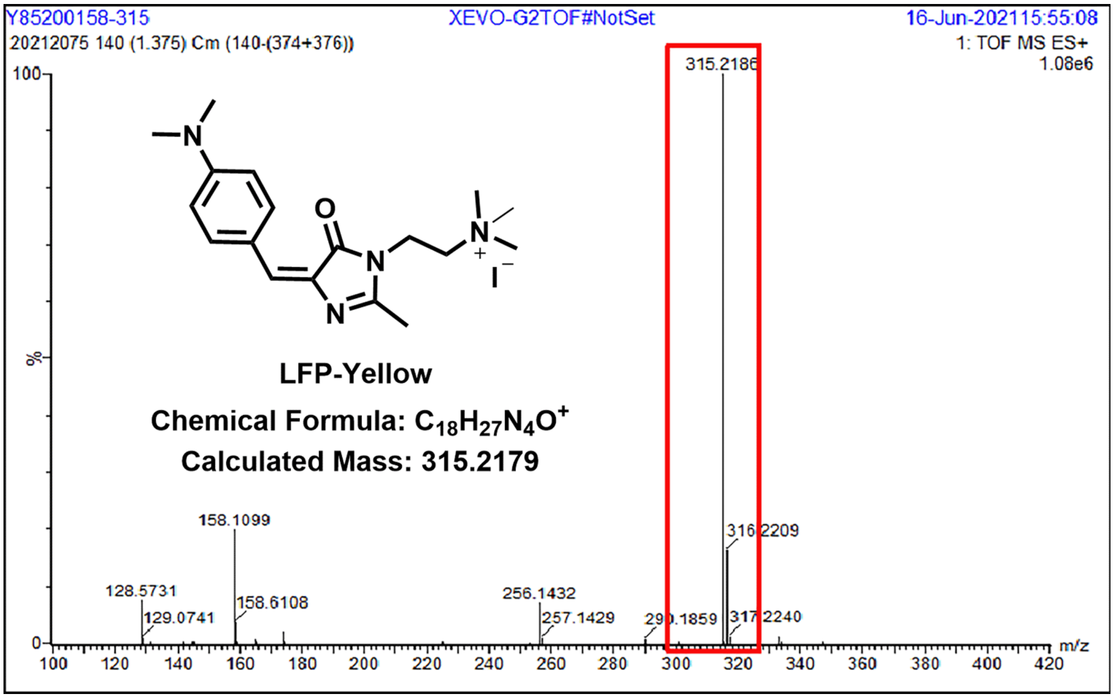

Figure S47. HRMS of LFP-Yellow.

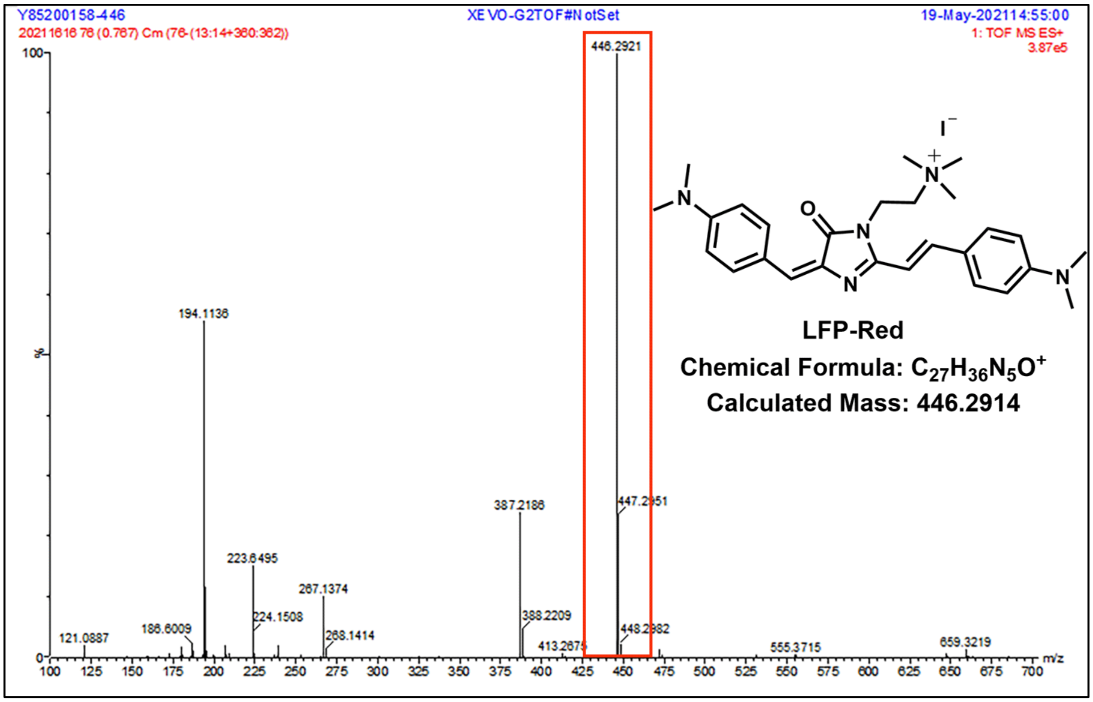

Figure S48. HRMS of LFP-Red.

4. Movies for development of LFPs

Movie S1 (separate file). Soaking method for the development of latent fingerprints.

Movie S2 (separate file). Portable ultrasonic atomizer for spraying.

Movie S3 (separate file). Spraying method with ultrasonic atomizer for the development of latent fingerprints.

5. References

1. Cai, L.; Li, H.; Yu, X.; Wu, L.; Wei, X.; James, T. D.; Huang, C. Green Fluorescent Protein GFP-Chromophore-Based Probe for the Detection of Mitochondrial Viscosity in Living Cells. *ACS Appl. Bio Mater.* **2021,** *4*, 2128-2134.
2. Klehs, K.; Spahn, C.; Endesfelder, U.; Lee, S. F.; Fürstenberg, A.; Heilemann, M. Increasing the Brightness of Cyanine Fluorophores for Single-Molecule and Superresolution Imaging. *ChemPhysChem* **2014,** *15*, 637-641.
3. D'Elia, V.; Materazzi, S.; Iuliano, G.; Niola, L. Evaluation and Comparison of 1,2-Indanedione and 1,8-Diazafluoren-9-one Solutions for the Enhancement of Latent Fingerprints on Porous Surfaces. *Forensic Sci. Int.* **2015,** *254*, 205-214.
4. BVDA Home Page. https://www.bvda.com/en/ind (accessed Oct 04, 2023).
5. Champod, C., Lennard, C. J., Margot, P., Stoilovic, M. *Fingerprints and Other Ridge Skin Impressions*, 2nd ed.; CRC Press, 2016.
6. Maltoni, D.; Maio, D.; Jain, A. K.; Feng, J. Fingerprint Analysis and Representation. In *Handbook of Fingerprint Recognition*, 3rd ed.; Springer Cham, 2022; pp 115-216.
7. Li, J.; Glover, J. D.; Zhang, H.; Peng, M.; Tan, J.; Mallick, C. B.; Hou, D.; Yang, Y.; Wu, S.; Liu, Y.; Peng, Q.; Zheng, S. C.; Crosse, E. I.; Medvinsky, A.; Anderson, R. A.; Brown, H.; Yuan, Z.; Zhou, S.; Xu, Y.; Kemp, J. P.; Ho, Y. Y. W.; Loesch, D. Z.; Wang, L.; Li, Y.; Tang, S.; Wu, X.; Walters, R. G.; Lin, K.; Meng, R.; Lv, J.; Chernus, J. M.; Neiswanger, K.; Feingold, E.; Evans, D. M.; Medland, S. E.; Martin, N. G.; Weinberg, S. M.; Marazita, M. L.; Chen, G.; Chen, Z.; Zhou, Y.; Cheeseman, M.; Wang, L.; Jin, L.; Headon, D. J.; Wang, S. Limb Development Genes Underlie Variation in Human Fingerprint Patterns. *Cell* **2022,** *185*, 95-112.
8. Pang, B. C. M.; Cheung, B. K. K. Double Swab Technique for Collecting Touched Evidence. *Leg. Med.* **2007,** *9*, 181-184.
